# Optimal programs of pathway control: dissecting the influence of pathway topology and feedback inhibition on pathway regulation

**DOI:** 10.1186/s12859-015-0587-z

**Published:** 2015-05-16

**Authors:** Gundián M de Hijas-Liste, Eva Balsa-Canto, Jan Ewald, Martin Bartl, Pu Li, Julio R Banga, Christoph Kaleta

**Affiliations:** 1 Bioprocess Engineering Group, Spanish National Research Council, IIM-CSIC, C/Eduardo Cabello 6, Vigo, 36208 Spain; 2 esearch Group Theoretical Systems Biology, Friedrich Schiller University Jena, Leutragraben 1,, Jena, 07743 Germany; 3 Simulation and Optimal Processes Group, Ilmenau University of Technology, P.O.Box 100565, Ilmenau, 98684 Germany; 4 Research Group Medical Systems Biology, Christian-Albrechts-University Kiel, Brunswiker Straße 10, Kiel, 24105 Germany

**Keywords:** Optimization, Gene expression, Optimal regulatory strategies

## Abstract

**Background:**

Adjusting the capacity of metabolic pathways in response to rapidly changing environmental conditions is an important component of microbial adaptation strategies to stochastic environments. In this work, we use advanced dynamic optimization techniques combined with theoretical models to study which reactions in pathways are optimally targeted by regulatory interactions in order to minimize the regulatory effort that is required to adjust the flux through a complex metabolic network. Moreover, we analyze how constraints in the speed at which an organism can respond on a proteomic level influences these optimal targets of pathway control.

**Results:**

We find that limitations in protein biosynthetic rates have a strong influence. With increasing protein biosynthetic rates the regulatory effort targeting the initial enzyme in a pathway is reduced while the regulatory effort in the terminal enzyme is increased. Studying the impact of allosteric regulation for different pathway topologies, we find that the presence of feedback inhibition by products of metabolic pathways allows organisms to reduce the regulatory effort that is required to control a metabolic pathway in all cases. In a linear pathway this even leads to the case where the sole transcriptional regulatory control of the terminal enzyme is sufficient to control flux through the entire pathway. We confirm the utilization of these pathway regulation strategies through the large-scale analysis of transcriptional regulation in several hundred prokaryotes.

**Conclusions:**

This work expands our knowledge about optimal programs of pathway control. Optimal targets of pathway control strongly depend on the speed at which proteins can be synthesized. Moreover, post-translational regulation such as allosteric regulation allows to strongly reduce the number of transcriptional regulatory interactions required to control a metabolic pathway across different pathway topologies.

**Electronic supplementary material:**

The online version of this article (doi:10.1186/s12859-015-0587-z) contains supplementary material, which is available to authorized users.

## Background

The control of metabolic pathways has been studied in-depth in the context of metabolic control analysis (MCA, [1,2]). This mathematical framework has a significant number of applications and variations [3-6]. While classical MCA has focused on steady states, several authors have used a dynamic optimization approach to study optimal dynamics in metabolic networks [7-13].

Wessely et al. [13] showed that, depending on protein costs, two distinct strategies for the control of metabolic pathways in a dynamic environment exist: sparse transcriptional regulation, in which only key enzymes of a pathway are transcriptionally regulated, and pervasive transcriptional regulation, in which each enzyme of a pathway is transcriptionally controlled. Pervasive transcriptional regulation represents the classical picture of pathway regulation where all the enzymes are regulated and is used to control metabolic pathways with a high protein cost. In contrast, sparse transcriptional regulation, which mostly targets initial and terminal steps of a metabolic pathway, is used for pathways with low protein costs. The existence of these two types of strategies can be explained by a trade-off between the cost of the enzymes catalyzing the reactions of the pathway, that is, the protein cost of the pathway, and response times that can be reduced by an exclusive transcriptional regulation of key steps in a pathway. This trade-off between protein cost and regulation was also used to explain the regulation of metabolism in *Saccharomyces cerevisiae* [14].

While Wessely et al. [13] considered the simple case of a linear metabolic pathway, this did not take into account that pathways often involve complex topologies of branching as well as diverging sub-pathways and are often controlled through feedback mechanisms exerted by the product of the pathway. Moreover, it was not taken into account how differences in the time-hierarchy between changes in enzyme concentration and resulting changes in metabolite concentration affect the optimal programs for pathway regulation that were identified. In another study, we analyzed how the capacity of the protein biosynthetic machinery in relation to the amount of protein to be produced influences the optimality of different types of activation programs for the enzymes of a metabolic pathway [12].

In this work, we investigate the influence of protein synthesis rates as well as feedback inhibition on optimal regulatory programs for the control of complex metabolic pathway topologies by means of advanced dynamic optimization techniques. We aim to draw general conclusions about optimal points of pathway control that are independent of the underlying kinetic parameters of the reactions constituting the pathway. Thus, we do not consider the example of a specific metabolic pathway but perform our analysis on a wide range of pathways with irreversible Michaelis-Menten-kinetics but varying kinetic parameters. Moreover, we need to exclude the influence of protein abundance on pathway control strategies since this factor is mostly independent of pathway structure and thereby occludes the influence of pathway structure on optimal regulatory strategies. Hence, we focus on transcriptionally sparsely regulated metabolic pathways which are characterized by a relatively small number of transcriptional regulatory interactions targeting key enzymes of a pathway.

In the first part of our work, we analyze how the introduction of a time-hierarchy between changes in metabolite and enzyme concentrations influences optimal targets of pathway control. We find that constraints on protein biosynthetic rates lead to an increase in the regulatory effort targeting the initial enzymes of pathways while the regulatory effort targeting the terminal enzyme is reduced. We confirm this pattern with an analysis of pathway regulation in prokaryotes with slow and fast protein biosynthesis.

In the second part, we analyze how the introduction of feedback inhibition by the product of a pathway in different pathway topologies influences optimal targets of pathway regulation. We find that introducing feedback inhibition reduces the regulatory effort that is required to control a metabolic pathway. In a linear pathway this even leads to the observation that a single regulatory interaction - the control of the terminal step of a pathway - is sufficient to precisely control the flux through the pathway. By analyzing optimal programs of pathway control for different strengths of feedback inhibition we find that there is an optimal value for the inhibitory constant at which the strength of the inhibitory is still high enough while minimizing the increased protein cost due to the inhibitory effect. In summary, our work provides important new insights into optimal strategies of pathway control and confirms the utilization of the proposed programs through the analysis of regulation in a large number of prokaryotic metabolic networks.

## Methods

### Mathematical problem formulation

The basic model used by Wessely et al. [13] consists of a linear metabolic pathway with four intermediates *X*_1_, …, *X*_4_ that are converted from a buffered substrate *S* into a product *P* via five enzymatic steps *e*_1_, …, *e*_5_. The enzymatic steps follow irreversible Michaelis-Menten kinetics. This approach has been used to provide important insights into general principles of the regulation of metabolism [8,12,15]. Though many pathways are comprised of a mixture of reversible and irreversible reactions, we only consider irreversible reactions due to the lower number of parameters which we need to consider for the sampling. Moreover, the validation is performed on metabolic pathways irrespective of the reversibility status of the constituting reactions. This indicates that the results we obtain for irreversible pathways also apply in the more general case for pathways also containing reversible reactions. Nevertheless, the consideration of reversibility is an important factor which we will consider in future work in particular in connection with the avoidance of intermediate accumulation and the facilitation of transitions between different product dilution rates.

Given a set of dilution values of the product of the pathway over a time-course, the aim of the optimization is to identify a time-course of the enzymes *e*_*i*_(*t*) that maintains the concentration of the product of the pathway within a given range and minimizes the objective function

(1) $$ {\fontsize{8 }{6}\begin{aligned} min \underbrace{\displaystyle\sum\limits_{i=1}^{n} \sigma\cdot e_{i}(0) \cdot t_{f}}_{\sigma \cdot J_{cost} = \displaystyle\sum\limits_{i=1}^{n} {cost}_{i}} + \underbrace{\displaystyle\sum\limits_{i=1}^{n} \int\displaylimits_{0}^{t_{f}} (e_{i}(t)-e_{i}(0))^{2} dt}_{J_{reg}=\displaystyle\sum\limits_{i=1}^{n} {reg}_{i}} \end{aligned}}  $$

where *n* corresponds to the number of enzymes of the pathway and *t*_*f*_ is the considered time frame.

The objective function has two components who’s individual contributions are adjusted through the weighting factor *σ*: the total protein cost *J*_*cost*_ and the regulatory component *J*_*reg*_ with the protein cost of each enzyme

(2) $$ {cost}_{i}= e_{i}(0) \cdot t_{f}  $$

and the regulatory effort of the individual enzymes

(3) $$ {reg}_{i}=\int\displaylimits_{t_{0}}^{t_{f}}(e_{i}(t)-e_{i}(0))^{2}dt.  $$

The regulatory effort is measured as the integral of the square deviation of the concentration of each enzyme from its initial value.

The control variables determine the system dynamics according to changes in the outflow of the product and have to obey constraints on the concentration of the product. Moreover, there are constraints on the concentrations of the pathway intermediates to prevent their accumulation to toxic levels. The importance of the two parts of the objective function can be adjusted by a weighting factor *σ* that is multiplied with initial enzyme concentrations. For small values of *σ*, initial enzyme concentrations have only low weight, that is, protein costs are low, while a high value of *σ* corresponds to high protein costs. For simplicity, we will assume throughout the manuscript that the determining factor in protein costs is the total abundance of a protein corresponding to the total amount of a protein present in a cell. For the complete mathematical formulation of the optimization problem see Additional file 1: Texts S1 and S2. By minimizing protein costs and the regulatory effort we account for two sometimes opposing forces that are strong determinants of bacterial fitness. While the production of protein is precisely adjusted to the need of the organism [16] also environmental uncertainties need to be taken into account. Thus, there is also a strong selective force to minimize transition times after a change in environmental conditions. Thereby, microorganisms are able to reduce their variance in fitness which can lead to growth advantages in fluctuating environmental conditions [17]. For instance, in *E. coli* it has been shown that metabolic fluxes show an adaptation toward the minimization of transition times [18]. In the context of our objective function, such a minimization of transition time is achieved by minimizing the regulatory effort – the amount of change required to adjust enzyme concentrations after a change in the required flux through the pathway.

In the optimization, the initial concentrations of the enzymes *e*_*i*_(0) and their time-courses *e*_*i*_(*t*) correspond to the control variables. By adjusting the time-courses of enzymes, the optimization procedure determines an optimal regulatory program that maintains product concentrations while the demand on pathway output is changed. *In vivo* this regulation can occur either on the transcriptional or translational level. However, we generally speak about transcriptional regulation since we focus on prokaryotes and in the model prokaryote *E. coli* there are, according to EcoCyc [19], only few regulatory interactions known that act on an exclusive translational level. Please note that, while dilution through growth can be considered as the major source of the dilution of a pathway product, we also use this formulation to account for events in which the concentration of the product needs to be adjusted. In a previous work we could show that we obtain similar results if we include constraints to adapt product concentrations but a more complex formulation of the dynamic optimization problem is required [13].

Optimizations were performed over 30 (arbitrary) time units. For randomized runs, kinetic parameters were uniformly drawn from the interval [0,2] as done previously in [13] and values for the dilution (*v*_*growth*_) from the interval [0.2, 0.8]. If not stated otherwise, 200 optimization runs with randomized parameter and dilution values were conducted for each analysis. For the consideration of limitations in protein biosynthetic rates, we additionally constrained concentrations changes of enzymes to a maximum of *m*:

(4) $$\begin{array}{@{}rcl@{}} \left| \frac{{de}_{i}(t)}{dt} \right| \le m & for & i=1:5  \end{array} $$

For more information, see Additional file 1: Text S2.

To test the robustness of our problem formulation, we investigated the influence of final time *t*_*f*_ and sampling approach for kinetic constants on results. We found that our conclusions for the basic optimization problem [13] remained the same when increasing final time (Additional file 1: Figure S2) and sampling kinetic constants from measured values (Additional file 1: Figure S3).

### Optimization approach

To determine optimal solutions, the original dynamic optimization problem is transformed into a non-linear programming problem (NLP) by means of the control vector parametrization approach [CVP] [20].

The non-linear character of the models and the presence of algebraic constraints may induce multimodality. Therefore, global NLP solvers are required. In the context of dynamic optimization, the importance and efficiency of global optimization techniques has been discussed in depth [21] [and references therein]. Recent works proposed hybrid global-local methods as an enhanced alternative for searching the global optimum [22,23].

In this work we use the enhanced scatter search method, eSS [23], which offers the possibility of using local deterministic methods from automatically selected initial points to enhance convergence rates to the global solution. Further details about numerical methods are presented in Additional file 1: Text S1.

### Validation of the optimal programs through data analysis

#### A genomic proxy for regulatory effort

The number of transcription factor binding sites as well as the number of different transcription factors controlling each gene are available only for a selected number of organisms such as *E. coli*. Therefore, we used the length of promoter regions as an indicator for the regulatory effort that is used to control a specific gene as done previously [24]. Thus, we assume that a more complex regulatory program (e.g. with a higher number of controlling transcription factors) that is used to control the expression of a gene will lead to longer promoter sequences. In contrast, if a gene is constitutively expressed or targeted only by few transcription factors, promoter sequences will be shorter. As described below, several lines of evidence strongly support this hypothesis.

Promoter lengths were determined as the length of the region upstream of the first gene of the operon to which the gene belongs (or upstream of the gene, if it does not belong to an operon) based on the annotation provided in the MicroCyc database [25]. Operon predictions were obtained from MicrobesOnline [26]. To take into account shared promoter regions, we considered the entire promoter region as contributing to the regulation of the gene if the preceding gene (in the direction of transcription) on the genome had the same direction of transcription. If the preceding gene had the opposite direction of transcription, the promoter lengths were considered to be equally shared between both genes.

To make promoter lengths comparable across organisms, we first made them comparable across the pathways within a single organism and subsequently across several organisms. To make promoter lengths comparable across pathways within a single organism, we subtracted the average promoter length of genes within this pathway from the promoter length of each gene. This is necessary since factors such as protein abundance have a strong influence on the regulatory effort targeted at a gene [13] and therefore on its promoter length. To make promoter lengths comparable across organisms, we divided them by the average promoter lengths of the non-metabolic genes of this organism (i.e. genes not annotated with a metabolic function). We did not consider negative promoter lengths, that is, cases in which coding regions of genes overlapped.

Several lines of evidence support that promoter lengths are a good indicator of the number of transcription factors regulating a gene and thereby of the regulatory effort targeting this gene.

First, most parts of bacterial genomes are made up of coding regions and non-functional elements of the genome are rapidly lost, in particular due to a bias towards deletions in bacterial genomes [27,28]. Since a higher number of transcription factors targeting a gene will require a longer promoter sequence and non-functional parts of a promoter sequence will be rapidly lost, the length of a promoter sequence indicates the number of transcription factors that target a specific gene. While also transcription factor binding sites within coding regions are known in *E. coli*, they make up only 12% of all known transcription factor binding sites in EcoCyc release 14.6 [19]. Moreover, if a transcription factor is only weakly binding a promoter sequence and we equate this with the assumption that this implies a marginal role in the regulation of the corresponding protein, there will be a bias toward the loss of this portion of the genome due to the above mentioned mutational bias. Again this leads to a tendency of a reduced promoter length reflecting a reduced regulatory effort targeted at the gene.

Second, we analyzed all promoter regions in *E. coli* and found a significant increase in the length of promoter regions with the number of known transcription factors controlling a gene (obtained from RegulonDB [29]). Classifying genes according to the number of transcription factors controlling them into genes with zero, one, two or more transcription factors (TFs) these increases are significant between all classes (Wilcoxon test p-values: 0 TFs vs. 1 TF, p-val =6.9·10^−5^, 1 TF vs. 2 TF, p-val =2.9·10^−9^, 2 TFs vs. >2 TFs, p-val =<10^−16^, see also Additional file 1: Figure S5).

Third, we find increased promoter lengths for initial and terminal enzymes in transcriptionally sparsely regulated metabolic pathways (Additional file 1: Figure S6A) and decreasing promoter lengths with pathway position in transcriptionally pervasively regulated metabolic pathways (Additional file 1: Figure S6B) across all organisms in the MicroCyc collection. Similar results have been obtained based on the number of transcription factors regulating each gene in *E. coli* previously [13].

Fourth, it has been reported previously that more abundant proteins have a higher number of transcriptional regulators controlling them in *E. coli* [13]. Therefore, we analysed the correlation between promoter lengths and protein abundance, measured by the codon adaptation index [30], across all organisms of the MicroCyc collection. After correcting for multiple testing using the Benjamini–Yekutieli procedure [31], we found that promoter lengths are significantly positively associated with protein abundance in 287 organisms, while we found a significant negative correlation only in 21 organisms. Hence, similar to the number of transcription factors, promoter lengths are also positively correlated with protein abundance.

#### Identification of transcriptionally sparsely and pervasively regulated metabolic pathways

Since our analysis is specific for sparsely regulated metabolic pathways, that is, pathways in which transcriptional control is not exerted to the same extent on each enzyme, we needed to determine sparsely regulated metabolic pathways in each organism. Since promoter lengths of genes allow us to assess the regulatory effort targeted at each enzyme, we classified metabolic pathways as sparsely regulated if the average length of promoter regions of genes along a linear chain of reactions within the pathway was below 60% of the promoter lengths of non-metabolic genes in this organism and as pervasively regulated otherwise. Results did not change for small changes in this threshold value. Non-metabolic genes were defined as those genes without a metabolic function according to the genome annotation in MicroCyc.

#### Determination of pathways

**Figure 1:**
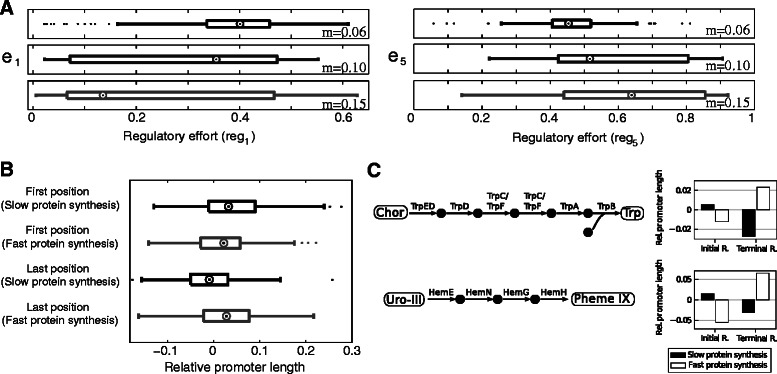
Influence of protein biosynthetic rates on positional pathway regulation.**(A)** Regulatory efforts (*r* *e* *g*_*i*_) at different pathway positions for constrained protein biosynthetic rates. A low value of *m* corresponds to slow protein biosynthesis while a high value corresponds to fast protein biosynthesis. Medians are indicated by circles. Complete boxplots are shown in Additional file 1: Figure S4. **(B)** The regulatory effort, measured as average relative lengths of promoter regions at different pathway positions has been determined for organisms with slow and fast protein synthesis rates. At the initial step of pathways the regulatory effort is increased for organisms with slow protein synthesis whereas it is increased at the terminal position for organisms with fast protein synthesis. Average promoter length for organisms with slow protein synthesis is depicted in black (154 organisms) and grey is used for 155 organisms with fast protein synthesis. **(C)** Relative average promoter lengths in selected pathways for organisms with slow and fast protein biosynthesic rates. The medians of relative average promoter length for tryptophan biosynthesis (upper panel, data from 83 organisms) and uroporphyrinogen-III biosynthesis (an intermediate of heme biosynthesis, lower panel, data from 114 organisms) are shown. Data is shown for the initial and terminal reactions. Protein names correspond to those of the catalytic pathway in *E. coli*. Abbreviations: Initial R., initial reaction; Terminal R., terminal reaction; Chor, Chorismate; Trp, L-Tryptophan; Uro-III, uroporphyrinogen-III; Pheme IX, protoheme IX.

Linear chains of reactions within a pathway were determined as described previously [12]. For the display of changes in relative average promoter lengths for organisms with slow and fast protein biosynthesis for specific pathways (Figure 1C), we selected individual pathways that were identified as sparsely regulated across a large number of organisms. We omitted data from organisms in which the corresponding pathway is organized in a single operon (and hence promoter lengths would be equal for all proteins). Statistical tests were performed using R [32]. All data used for validation can be found in Additional file 2.

#### Mapping of post-translationally regulated reactions

To estimate post-translational regulation, post-translational modification (PTM) sites of proteins were retrieved from the data base dbPTM [33], which contains information about all kinds of modification sites across all domains of life. Because only for a small number of proteins experimentally validated post-translational modifications sites are available, those were combined with predicted modification sites listed in the same database. The UniProt identifiers of the investigated enzymes were used to map the modifications sites to the reactions of the metabolic pathways. Data on post-translational modifications can be found in Additional file 3.

## Results

### Influence of protein biosynthetic rates on pathway regulation

In a previous work we showed that the protein biosynthetic rate of an organism has a strong influence on activation strategies of metabolic pathways [12]. The protein biosynthetic rate of an organism corresponds to the rate at which proteins can be produced. As we’ve shown in a previous work, the genomic copy number of ribosomal RNAs is strongly associated to the protein biosynthetic capacity of an organism [12]. Therefore, we analyzed optimal targets of pathway control with an additional constraint on the rate of change of enzyme concentrations for different protein biosynthetic rates (see Additional file 1: Text S2 for the problem formulation). We analyzed the regulatory effort in a linear pathway (without feedback inhibition) for three different protein biosynthetic rates across 150 models with random kinetic parameters and dilution values. We observed that a high protein biosynthetic rate leads to a decrease of the regulatory effort at the first reaction of the pathway (Wilcoxon test *p-value* =1.27·10^−5^ between regulatory effort for m=0.06 and m=0.15) and a concomitant increase at the terminal step of the pathway (Wilcoxon test *p-value* <10^−16^, between regulatory effort for m=0.06 and m=0.15, Figure 1A). This observation also holds when considering differences in the time-scales between metabolite, transcriptional and growth dynamics (Additional file 1: Figure S7).

Thus, the protein biosynthetic rate has an influence on the optimal regulatory effort at each pathway position. By decreasing protein biosynthetic rates, we limit the amount of change in the individual enzymes while there is no constraint on the amount of change in metabolite concentrations. In consequence, in relative terms the effects of changes in protein concentrations on metabolite concentrations propagate faster through the network. Hence, also the control of the first enzyme on the flux through the entire pathway becomes more immediate. In contrast, if protein concentrations can be adapted more rapidly, also a control of the terminal enzymes of pathways is of advantage since adjusting the concentration of the terminal enzyme of a pathway allows for an immediate change of pathway output while for slow changes in protein concentrations a part of this function can be taken over by the initial enzyme.

We investigated whether we could observe this pattern of regulation *in vivo*. To this end, we analysed the regulatory effort at different pathway positions across several hundred prokaryotes with different protein biosynthetic capacities. As a proxy for the regulatory effort that is exerted on each enzyme, we used the length of non-coding regions upstream of each gene (see Methods).

We ordered organisms according to the copy number of ribosomal RNAs in their genome and grouped them into organisms with low and high protein biosynthetic rates correspondingly (lower and upper 50% of organisms). Subsequently, we determined the average relative length of promoter regions for the initial as well as terminal enzymes in sparsely regulated metabolic pathways (Figure 1B). In a direct comparison of relative promoter lengths between organisms with low and high protein biosynthetic rates we found a significant decrease at the beginning of pathways (Wilcoxon test *p-value* =1.2·10^−2^) and a significant increase at the end of pathways (Wilcoxon test *p-value* =3.56·10^−5^). We performed this analysis in more detail for two pathways amongst the transcriptionally sparsely regulated pathways in the largest number of organisms: tryptophan biosynthesis and protoheme IX biosynthesis, an intermediate of heme biosynthesis. In both cases we observed a decrease in the regulatory effort targeting the first reaction and an increase in the last reaction of the corresponding pathways for organisms with faster protein biosynthetic rates (Figure 1C). Thus, as predicted by the optimization, there is a shift in the regulatory effort from the first to the terminal enzyme with an increasing protein synthesis rate.

These results also provide an explanation why we observed that the increase in the frequency of transcriptional regulatory interactions in pathways in *E. coli* is stronger at the end of metabolic pathways than at the beginning [13], since *E. coli* has a high copy-number of rRNAs in comparison to other prokaryotes.

### Feedback inhibition in linear pathways

**Figure 2:**
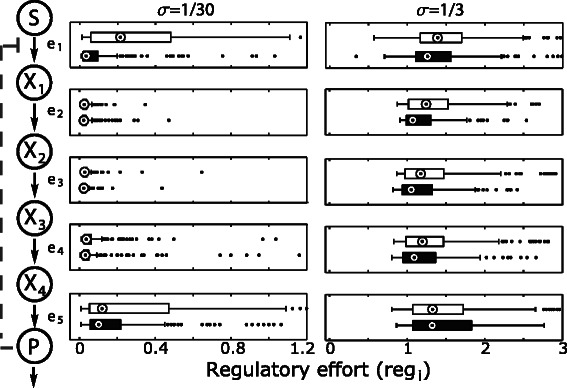
Regulatory efforts (*r* *e* *g*_*i*_), measured as change in enzyme concentrations, for different weights of initial enzyme concentrations. Optimizations without inhibition depicted in white, runs with inhibition depicted with black boxes. Complete boxplots are shown in Additional file 1: Figures S11 and S12.

A frequently encountered mechanism in the control of metabolic pathways is a feedback inhibition of the initial enzyme by the product [34-36]. To study this mechanism, the problem was modified by introducing a competitive inhibition of the first enzyme *e*_1_ of the pathway by the product *P* (Figure 2):

(5) $$ \upsilon_{1}(t)=\frac{k_{cat,1}\cdot e_{1}(t)\cdot s(t)}{s(t)+K_{m}\left(1+\frac{p(t)}{k_{r,1}}\right)}   $$

with *k*_*r*,1_ corresponding to the strength of the feedback inhibition. For increasing values of *k*_*r*,1_ inhibition is weaker and for decreasing values it is stronger.

**Figure 3:**
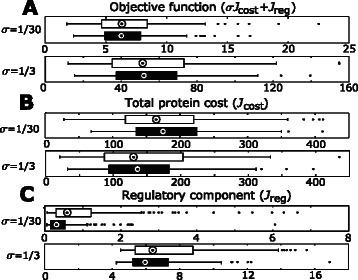
Influence of inhibition on the objective function. The values of the two components of the **(A)** objective function: **(B)** total protein cost (*σ*·*J*_*cost*_) and **(C)** regulatory component (*J*_*reg*_), are compared for cases with (black bars) and without inhibition (white bars). Medians are denoted by circles.

In a first step, we analyzed how the introduction of a feedback inhibition influences the individual components of the objective function, the regulatory effort as well as the initial enzyme concentrations. We performed these comparisons for two different values of the weighting factor *σ* using unit inhibitory constants (Figure 3).

We did not observe a significant change in the objective function values after introduction of the inhibition across both weights for initial enzyme concentrations (Figure 3A). The contribution of the regulatory component *J*_*reg*_ (deviation from the initial enzyme concentrations) was significantly decreased across all cases. This was mostly marked for a low weight of initial enzyme concentrations (Wilcoxon test *p-value* =8.92·10^−11^) while it was not as strong for a high weight of initial enzyme concentrations (Wilcoxon test *p-value* =2.9·10^−2^). Though we observed a tendency of the initial concentrations of enzymes to increase with introduction of the feedback inhibition, this increase was not significant (Wilcoxon test *p-value* >0.1 for each case).

Analysing changes in the regulatory effort targeting individual enzymes (Figure 2), we found a strong decrease in the first enzyme. This change was strongest (Wilcoxon test *p-value* <10^−16^) for a low weight of initial enzyme concentrations. In consequence, particularly for pathways with lowly abundant proteins (low *σ*-values) the introduction of a feedback inhibition appears to relieve the requirement of a control of the first enzyme. Thus, due to the presence of the feedback inhibition, the flux through the entire pathway can be controlled through transcriptionally regulating the terminal step of the pathway. These results substantiate the observation that flux through a pathway can be controlled much more precisely through a regulation of the terminal enzyme. This is in contrast to the classical biochemical picture of pathway control in which the first enzyme has been considered the most relevant [35]. However, please note that since our optimization approach focuses on optimal responses to changes in product consumption while assuming a constant supply of the substrate of the pathway, the relevance of the individual enzymes might also differ if we consider changes in substrate concentrations.

For a high weight of initial enzyme concentrations the reduction in the regulatory effort was reduced but still significant (Wilcoxon test *p-value* =2.8·10^−4^). Thus, while protein abundance has an influence on the strength of the reduction, it is significant across all the considered cases. This is in line with previous observations that the utilization of post-translational regulation is not influenced by the abundance of proteins [13].

**Figure 4:**
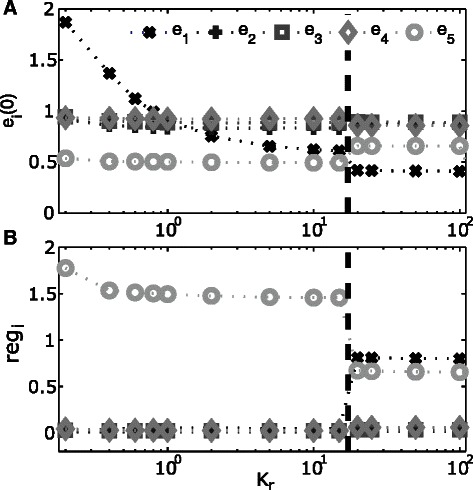
Influence of regulatory strength (*k*_*r*,1_).**(A)** Initial enzyme concentrations for the different values of the regulatory constant *k*_*r*,1_ **(B)** Regulatory efforts (*r* *e* *g*_*i*_) for different values of *k*_*r*,1_. Please note the logarithmic scale of the x-axis. The dashed line indicates the value of the inhibitory constant *k*_*r*,1_ at which transcriptional regulation is switching from a sole regulation of the terminal enzyme to a regulation of initial and terminal enzyme.

As discussed above, the introduction of a feedback inhibition reduces the efficiency of the first enzyme. Thus, higher concentrations of *e*_1_ are required to achieve the same flux (cf. Additional file 1: Figure S10). In consequence, also initial enzyme concentrations are increased. To further investigate this effect, we repeated the optimization with different values of the inhibition constant *k*_*r*,1_ between [0, 100]. We observed that an increase of *k*_*r*,1_ had a strong effect on the initial concentration of *e*_1_ (Figure 4A). However, with an increasing value of *k*_*r*,1_ also the strength of the inhibition is reduced and thus also its control over the flux through the first enzyme. In consequence, if *k*_*r*,1_ is larger than a specific threshold value, the first enzyme is again under transcriptional control (Figure 4B). These two opposing trends result in the optimality of a specific value of *k*_*r*,1_ at which the inhibition is still strong enough to exert a regulatory effect on the first enzyme, while the costly increase of the concentration of *e*_1_ to maintain pathway flux is minimized (Figure 4A).

### Optimal regulatory programs for complex pathway topologies

Since metabolism often involves more complex topologies than the simple linear pathway considered above, we investigated optimal regulatory programs in two more complex pathway topologies: a converging pathway leading from two substrates to a product and a diverging pathway producing two distinct products from a single substrate (See Additional file 1: Text S3 for the problems formulations).

#### Optimal regulatory programs in pathways with a converging reaction

**Figure 5:**
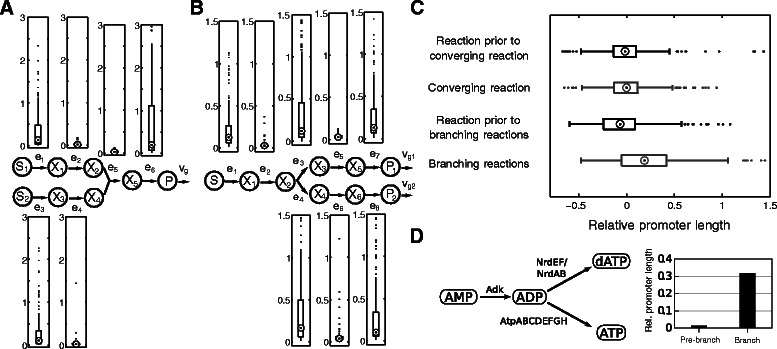
Optimal regulatory programs in complex pathways.**(A)** Regulatory efforts (*r* *e* *g*_*i*_, y-axis) for a converging pathway. **(B)** Regulatory efforts (*r* *e* *g*_*i*_, y-axis) in a diverging pathway. Complete boxplots are shown in Additional file 1: Figures S19 and S20. **(C)** Validation of optimal regulatory strategies. The first row display the average length of promoter regions prior to a converging reaction in pathways and the second row the average length of promoter regions in enzymes catalyzing the converging reaction (data for 248 organisms). The third row displays the average relative promoter lengths prior to a pathway branch and the fourth row the average relative promoter lengths of enzymes of the branching reactions (data for 142 organisms). For clarity, the distribution is cut at the value of 1.5 in the positive quadrant of the x-axis. **(D)** The median of relative average promoter lengths at a branching reaction in adenosine nucleotide biosynthesis (data from 77 organisms) are shown. “Pre-branch” corresponds to promoter lengths of proteins catalyzing the reaction before the branch (Adk) and “branch” to the promoter lengths of proteins catalyzing the subsequent reactions.

In a first setup, we considered a pathway in which two substrates are converted into a common product which is drained through *ν*_*growth*_ (Figure 5A). For the individual steps, we assumed irreversible Michaelis-Menten-kinetics as above.

The regulatory effort in different pathway positions is displayed in Figure 5A. While most of the regulation was observed in the initial and terminal steps of the pathway, there was almost no regulation at intermediate positions (*e*_2_, *e*_4_, *e*_5_).

These results show that in the case of a metabolic pathway with two convergent branches, it is still sufficient to control both the initial and the terminal steps of the pathway and there is no regulation around the converging step. Thus, the flux through the entire pathway, also after the branch, can be controlled by the initial enzymes of the individual pathway branches.

To verify the prediction that there are no differences in the regulatory effort around a convergent branch, we analyzed the average length of promoter regions of proteins catalyzing the corresponding reactions across our collection of prokaryotes. In confirmation of the optimization results, we did not observe a change in the length of promoter regions between reactions prior to a converging reaction and the converging reaction itself (Figure 5C, Wilcoxon test *p-value* =0.29).

#### Optimal regulatory programs in pathways with a divergent branch

In a second step, we analyzed regulatory programs to control a metabolic pathway that diverges into two distinct branches. We assumed that the products of the individual branches are drained with two different rates, *ν*_*g*1_ and *ν*_*g*2_, to account for potential differences in their production, for instance, when concentrations need to be adapted independently. Initially, we considered a pathway with eight reactions with irreversible Michaelis-Menten kinetics (Figure 5B).

We found that apart from a regulation in the initial and terminal reactions, also frequently the enzymes after the pathway branch (*e*_3_, *e*_4_) were regulated (Figure 5B). In contrast, upstream of the branch, we did not observe much regulation. Hence, a regulation after the branch appears to provide a better control of flux through the entire pathway than before the branch. This has important consequences on pathways in which the product of the pathway is substrate to further pathways since, in principle, no transcriptional control of the terminal step of the pathway would be required in such a case.

To test the predictions of the optimization, we analyzed the relative length of promoter regions of enzymes catalyzing reactions before and after branching reactions in the metabolic networks of our prokaryote collection. We found that in sparsely regulated metabolic pathways there was a significant increase in the length of promoter regions of enzymes after the branching reaction compared to the preceding reactions (Figure 5C, Wilcox test *p-value* =6.96·10^−11^). A specific example for the regulatory effort before and in a set of branching reactions in adenosine nucleotide biosynthesis is shown in Figure 5D.

#### Feedback inhibition over different positions of the pathway

**Figure 6:**
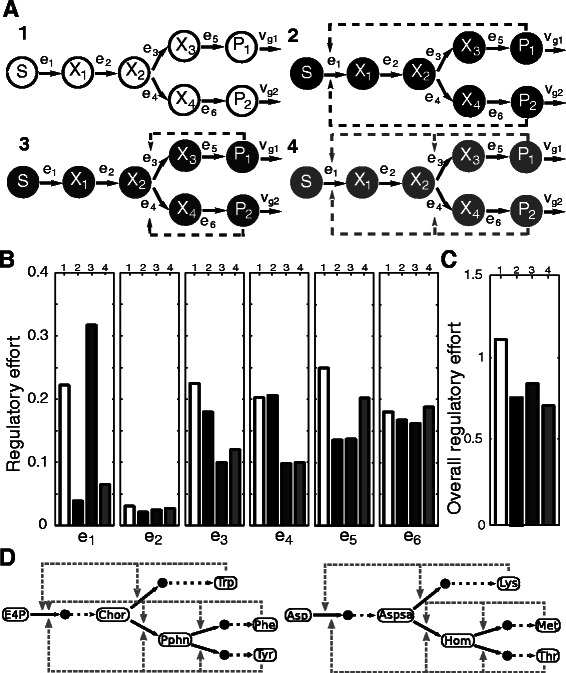
Feedback inhibition at different positions of the pathway.**(A)** Schematic representation of the different considered pathway configurations. **(B)** Median regulatory effort in each enzyme for different topologies of feedback inhibition. Runs without inhibition are depicted in white. Boxplots for the complete range of values are presented in Additional file 1: Figures S21-S24. **(C)** Overall regulatory effort measured as the sum of the median values for all the considered cases. **(D)** Example pathways from aromatic amino acid (upper pathway) as well as lysine, methionine and threonine biosynthesis (lower pathway) in which the identified regulatory strategies are utilized in *E. coli*. Gene names have been omitted for clarity. Dotted arrows correspond to lumped sequences of reactions, grey dashed arrows correspond to feedback inhibition. Figure adapted from the corresponding super-pathways in EcoCyc [19]. Abbreviations: E4P, D-erythrose 4-phosphate; Chor, chorismate; Trp, L-tryptophan; Pphn, prephenate; Phe, L-Phenylalanine; Tyr, L-tyrosine; Asp, L-aspartate; Aspsa, L-aspartate 4-semialdehyde; Lys, L-lysine; Hom, L-homoserine; Met, L-methionine; Thr, L-threonine.

Since we did not observe any regulation in the intermediate enzymes, we considered the impact of feedback inhibition in a reduced network in which *e*_7_ and *e*_8_ were removed (Figure 6A). Three different cases of feedback inhibition were considered for unit inhibitory constants (Figure 6A): 1) an inhibition of the initial step of a pathway by the two products (panel 2 in Figure 6A), 2) an inhibition of the branching enzymes *e*_3_ and *e*_4_ by the products *P*_1_ and *P*_2_, respectively, (panel 3 in Figure 6A) and 3) a combination of the two previous cases (panel 4 in Figure 6A) which corresponds to a nested feedback inhibition [37].

In general, we observed a decrease in the regulatory effort for enzymes targeted by feedback inhibition (Figure 6B). In the case of a feedback inhibition from the products of a pathway on the first step of the pathway, we observed a drastic decrease in the regulatory effort at this position. This decrease was not as strong when the products of the pathway additionally influenced the reactions after the branchpoint (*e*_3_ and *e*_4_).

The introduction of different types of feedback inhibition reduced the overall regulatory effort that is required to control the flux through the pathway across all different cases (Figure 6C). We observed the strongest decrease in the regulatory effort required for the case in which the products inhibited the initial reaction as well as the reaction after the branching point. In consequence, in principle a transcriptional control of the terminal steps of the individual pathway branches would be sufficient for a full control of the flux through the pathway. Thus, the introduction of feedback inhibition allows the reduction of the required number of transcriptional regulatory control points from five to two. The optimality of this pattern of feedback inhibition is exemplified by its implementation in several pathways in *E. coli* metabolism including aromatic amino acid biosynthesis, the combined metabolism of lysine, methionine as well as threonine, branched chain amino acid biosynthesis and purine biosynthesis (Figure 6D).

### Post-translational regulation reduces the transcriptional regulatory effort targeted at enzymes

As our optimization results have shown, post-translational regulation in general reduces the regulatory effort that is required to control the flux through a metabolic pathway. To test this assumption, we investigated the association between the occurrence of post-translational regulation and the length of promoter regions. As reference for post-translational regulation across our organism set, we used the dbPTM data base [33] that contains a large number of experimentally verified and predicted sites of post-translational modifications across all domains of life. We used these protein modifications as a reference for post-translational regulation since large-scale information about feedback inhibition in metabolism is only available for very few organisms.

**Figure 7:**
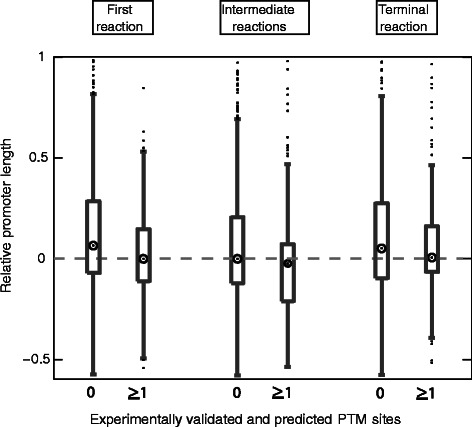
Interplay between transcriptional and post-translational regulation. Promoter lengths have been determined across all considered organisms for different pathway positions depending on the number of post-translational modification sites (PTM sites) of enzymes. The density distribution of relative promoter lengths is shown. For each pathway position the left violin plot indicates promoter lengths for enzymes without a post-translational modification site and the right violin plot relative promoter lengths for enzymes with at least one post-translational modification site. Circles denote the median of the distribution. In the positive quadrant violin plots are only shown for values up to one for clarity.

For transcriptionally sparsely regulated metabolic pathways, we compared how the presence of post-translational regulation influenced promoter lengths at different pathway positions (Figure 7). We observed for all pathway positions that promoter lengths were significantly shorter if an enzyme is post-translationally regulated (Wilcoxon test for initial reactions: p-val =8.6·10^−8^, intermediate reactions: p-val ≤2.2·10^−16^, terminal reactions: p-val =1.9·10^−3^). The relative difference in promoter lengths was largest for initial reactions. Thus, the in vivo validation confirmed that post-translational regulation reduced the amount of regulatory effort targeted at an enzyme. Beyond the consideration of optimal programs for pathway regulation this result also represents a very interesting aspect considering that it is usually assumed that post-translational and transcriptional regulation act on completely different time-scales [38]. Our optimization predicts and the validation shows that despite this separation of time-scales, both types of regulation appear to be interchangeable to some extent.

## Conclusions

In this work we used simplified models of metabolic pathways to study the influence of protein synthesis rates as well as feedback inhibition on optimal programs for the control of metabolism. Considering constraints on protein synthesis rates, we observed that a slower protein synthesis rate entails a reduction of the regulatory effort in the terminal step with a concomitant increase at the beginning of pathways. Through an analysis of the regulatory effort in pathways in a large number of prokaryotes, we could confirm these predictions. Considering individual organisms, these results also imply that environmental conditions might influence the optimal strategy to control a metabolic pathway. Since protein biosynthetic rates are strongly influenced by environmental conditions [39], there might even be shifts in the relevance of transcriptional control of individual enzymes between conditions that allow for slow or fast growth.

Analyzing the relevance of post-translational regulation with feedback inhibition as an example for pathway control, we found that post-translational regulation allowed to decrease the regulatory effort that is required to control a pathway for a wide variety of pathway topologies. We confirmed these results through the analysis of the interplay between transcriptional and post-translational regulation in our organism set. As a result, for the full control of a linear metabolic pathway it is sufficient to only regulate the terminal enzyme if the initial enzyme is inhibited by the product of the pathway. For branching pathways, we found that the optimal control points we identified resembled known patterns of feedback inhibition implemented in *E. coli* metabolism. These results are of particular importance in the context of gene expression analysis. There it is often implicitly assumed that changes in the expression of enzymes are equally important across all enzymes of a pathway. As our results show and additionally to the influence of allosteric regulation, transcriptional control favours those enzymes that provide better control of the flux through a pathway. Thus, depending on the topology of the metabolic network, changes in the expression of key enzymes are likely much more relevant than those of other enzymes of the same pathway. In the extreme case, a transcriptionally sparsely regulated metabolic pathway might only show changes in the expression of the terminal enzyme which will be certainly missed by conventional gene set enrichment analyses [40]. However, though a weighting of genes in gene set enrichment analyses can be considered [41], there exists so far no method that takes into account the relevance of enzymes for the regulation of metabolic fluxes in the context of this kind of methods.

## Additional files

Additional file 1
**Further details of optimal programs of pathway control.** The Additional file 1 presents a more detailed description of the numerical approaches used in this work as well as detailed model formulations for the different cases.

Additional file 2
**Supplemental Data.**

Additional file 3
**Data on post-translational modifications.**
