# Supplementary material for: Optimal programs of pathway control: dissecting the influence of pathway topology and feedback inhibition on pathway regulation
Source: BMC Bioinformatics. 2015 May 16;16:163. doi: 10.1186/s12859-015-0587-z (PMC4433072; doi:10.1186/s12859-015-0587-z)
Supplement: Additional file 1 — Further details of optimal programs of pathway control. The Additional file 1 presents a more detailed description of the numerical approaches used in this work as well as detailed model formulations for the different cases. [file 12859_2015_587_MOESM1_ESM.pdf]

Optimal programs of pathway control: Dissecting the influence of pathway topology and feedback inhibition on pathway regulation
Additional File 1

Gundián M. de Hijas-Liste[1], *Eva Balsa-Canto*[1], *Jan Ewald*[2], *Martin Bartl*[2,3], *Pu Li*[3], *Julio R. Banga*[1] *and Christoph Kaleta*[4,2]

[1]Bioprocess Engineering Group, Spanish National Research Council, IIM-CSIC, Eduardo Cabello 6, 36208 Vigo, Spain
[2]Research Group Theoretical Systems Biology, Friedrich Schiller University Jena, Leutragraben 1, 07743 Jena, Germany
[3]Simulation and Optimal Processes Group, Ilmenau University of Technology, P.O.Box 100565, 98684 Ilmenau, Germany
[4]Research Group Medical Systems Biology, Christian-Albrechts-University Kiel, Brunswiker Straße 10, 24105 Kiel, Germany

---

S1 Details on the implementation of the control vector parameterization approach

To solve dynamic optimization problems the most common used approaches can be grouped in two main categories: 1) Indirect approaches, based on Pontryagins maximum principle, this results in a two or multi-point boundary problem, in the presence of constraints that must be solved to state and co-state variables; 2) Direct approaches, like the complete parameterization (CP, Biegler *et al.*, 2002), multiple shooting (MS, Bock and Plitt, 1984) or control vector parameterization (CVP, Vassiliadis *et al.*, 1994), which transform the original optimization problem into a non-linear programming (NLP) using discretization and approximation for the control variables or for control and state variables. Theses approaches differ in the number of decision variables, the presence or not of parametrization related constraints and in the need or not of boundary value problem solver. Solving large-scale dynamic optimization problems with CP or MS can be computationally expensive. The CVP approach allows one to solve these problems abiding dealing with large NLPs and extra junction constraints.

Control Vector Parametrization

In the control vector parametrization approach (CVP, Vassiliadis *et al.*, 1994) the dynamic optimization problem is converted into a non-linear programming (NLP) problem with dynamic and algebraic constraints through the division of the time horizon into ρ time intervals and the approximation of the control variables (e_i) by low-order polynomials in each of those intervals. The resulting NLP has to be solved using a suitable NLP solver. It should be remarked that an IVP (initial value problem) solver will be necessary to handle dynamic constraints. Here RADAU5 (Hairer and Wanner, 1996), an implicit Runge-Kutta method, was used. For all examples the integration tolerances (absolute and relative) were set to 10^{-7}.

To study the influence of protein biosynthetic rates the control variables are approximated using piece-wise linear interpolation with varying length elements (PWL-v). The solution is obtained by 14 variable length linear elements (i.e. 89 decision variables).

To determine the impact of allosteric regulation on different pathway configurations, the control variables were approximated by piece-wise constant interpolation with elements of fixed length (t_f/ρ). A coarse approximation of the optimal solution is first obtained using a low discretization level (ρ). To efficiently achieve smooth control profiles a mesh refining approach (Balsa-Canto *et al.*, 2001) is subsequently used. In Table S1 the initial discretization level for each case and the NLP solved in each case is summarized.

Problem label	Controls	$\rho_{initial}$	NLP_{final}		
			CVP	Decision variables	Constraints
Linear pathway					
With and Without inhibition	5	20	160	800	3
Convergent Branch	6	20	160	960	3
Divergent Branch					
No inhibition and eight enzymes	8	15	120	960	3
All cases with six enzymes	6	15	120	720	3

Table S1: **Summary of the CVP scheme used for each example.** Problem label corresponds to the short name given to each of the examples in the main text; Controls, correspond to the control variables enzymes (e).

Nonlinear programming methods

The NLP obtained from applying direct approaches is frequently multimodal due to the presence of nonlinear or bi-linear dynamic constraints. Therefore, local deterministic methods for the solving these NLPs may fail to converge or converge to local solutions if the initial control profiles are far away from the global optimum. Which methods are the most adequate to solve these problems has been discussed in-depth in the literature (cf. Banga *et al.*, 2005, and references therein).

Recent works have suggested that from the different alternatives to search for the global optimum (deterministic, stochastic and hybrid optimization methods) hybrid global-local methods offer the best compromise between robustness and efficiency in the solution of this type of problems (Balsa-Canto *et al.*, 2005; Egea *et al.*, 2009).

In this work, a population based method, the enhanced scatter search method, eSS (Egea *et al.*, 2009) was used, this method allows to call local deterministic methods from automatically selected initial points to enhance convergence rates to the global solution. The mesh-refining approach is subsequently applied using FSQP, an efficient sequential quadratic programming method developed by Zhou et al, which ensures the convergence to a KKT point (Zhou *et al.*, 1997).

S2 Linear pathways

Mathematical formulation of the optimization problem

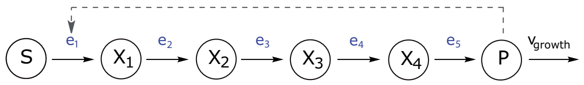

Figure S1: Representation of the linear pathway with a feedback inhibition of the first enzyme by the product of the pathway (dashed line). The basic model used by (Wessely *et al.*, 2011) does not include the feedback inhibition.

The mathematical formulation of the optimization problem in a linear pathway (Figure S1) as considered by Wessely *et al.* (2011) is given in the following. Note that the model has arbitrary units.

Find $\mathbf{e}(t)$ over t ϵ $[t_0,t_f]$ to minimize:

$$min \underbrace{\sum_{i=1}^{5} \sigma \cdot e_i(0) \cdot t_f}_{J_{cost}=\sum_{i=1}^{5} cost_i} + \underbrace{\sum_{i=1}^{5} \int_{t_0=0}^{t_f=30} (e_i(t) - e_i(0))^2 dt}_{J_{reg}=\sum_{i=1}^{5} reg_i} \tag{1}$$

Subject to the system dynamics:

$$\begin{aligned} \frac{ds(t)}{dt} &= 0 \\ \frac{dx_1(t)}{dt} &= \nu_1(t) - \nu_2(t) \\ \frac{dx_2(t)}{dt} &= \nu_2(t) - \nu_3(t) \\ \frac{dx_3(t)}{dt} &= \nu_3(t) - \nu_4(t) \\ \frac{dx_4(t)}{dt} &= \nu_4(t) - \nu_5(t) \\ \frac{dp(t)}{dt} &= \nu_5(t) - \nu_{growth}(t) \end{aligned} \tag{2}$$

$$\upsilon_{growth}(t) = \begin{cases} d_1 & \text{if } t < 10 \\ d_2 & \text{if } 10 \le t \le 20 \\ d_3 & \text{if } 20 \le t \le 30 \end{cases} \tag{3}$$

Where:

$$\upsilon_1(t) = \frac{k_{cat,1} \cdot s(t)}{s(t) + K_m \left(1 + \frac{p(t)}{k_{r,1}}\right)} \cdot e_1(t) \tag{4}$$

$$\upsilon_i(t) = \frac{k_{cat,i} \cdot x_{i-1}(t)}{K_{m,i} + x_{i-1}(t)} \cdot e_i(t) \tag{5}$$

And in the case without inhibition the flux though the initial reaction ($\upsilon_1(t)$) is:

$$\upsilon_1(t) = \frac{k_{cat,1} \cdot s(t)}{K_{m,1} + s(t)} \cdot e_1(t) \tag{6}$$

And the following additional constraints and initial conditions:

$$0.8 \le p(t) \le 1.2 \tag{7}$$

$$x_1(t) + x_2(t) + x_3(t) + x_4(t) \le \Omega \tag{8}$$

$$e_i(t) \ge 0 \tag{9}$$

with:$k_{cat,i}$ and $K_{m,i}$ ϵ[0,2], $[d_1, d_2, d_3]\epsilon[0.2, 0.8]$, $\Omega = 4$, $s(t_0) = 1$, $x_i(t_0) = 1$, $p(t_0) = 1$ and $k_{r,1} = 1$.

The aim of the optimization is to identify a time-course of the enzymes $e_i(t)$ (which represent the control variables of the optimization problem) that minimizes a objective function (Eq. 1) which has two parts: the total protein cost (J_{cost}) and the regulatory component (J_{reg}). The control variables determine the system dynamics (Eq. 2) according to changes in the outflow of the product (Eq. 3) and have to obey constraints on the concentration of the product (Eq. 7) as well as the concentration of the intermediate metabolites (Eq. 8).

In the objective function (Eq. 1), we minimize the sum of the integral of the initial enzyme concentration $e_i(0)$ over time multiplied with the weighting factor σ and the square of the deviation of the enzyme concentration $e_i(t)$ from its initial value $e_i(0)$. With the weighting factor σ we can adjust the importance of initial enzyme concentrations. Thus, for small σ values, initial enzyme concentration has a stronger impact than changes in enzyme concentration. In a biological context, a small σ value corresponds to proteins that are present in low abundances since their corresponding fitness cost is low. A high σ value, in contrast, corresponds to proteins that are present in high amounts and thus incur a high fitness cost in comparison to lowly abundant proteins.

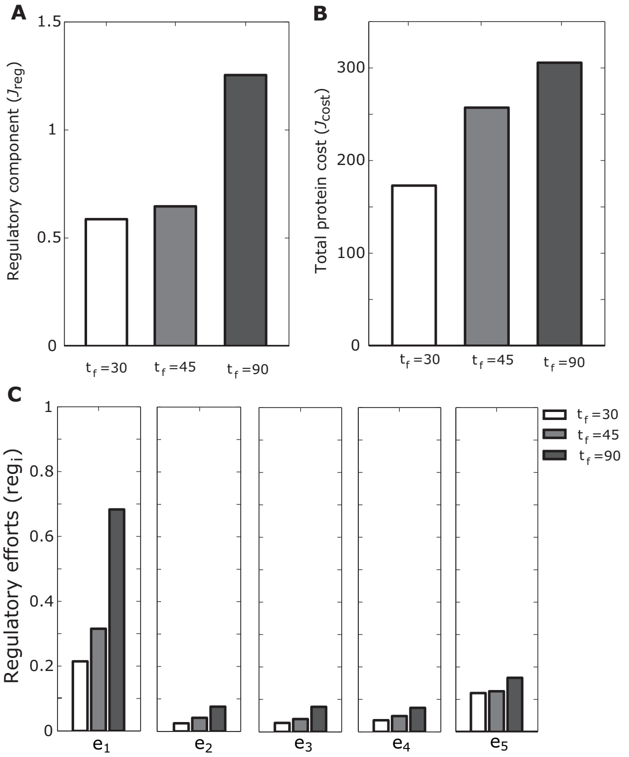

Figure S2: **Influence of final time on pathway regulation.** Median values of (**A**) regulatory component (J_{reg}) , (**B**) total protein cost (J_{cost}) and (**C**) individual regulatory efforts (reg_i), measured as absolute change in enzyme concentrations, at different pathway positions for three different final times $t_f = 30, 45, 90$. For simulations, switching times of v_{growth} were adapted to 1/3 and 2/3 of t_f accordingly. For different final times, we did observe an increase in the regulatory component (J_{reg}) and an increase of the total protein cost (J_{cost}). Analysing changes in the regulatory effort targeting individual enzymes, we found that beside differences in the relative values of regulatory efforts, initial and terminal enzymes were principal targets of regulation. The increase in the regulatory effort targeted at the first enzyme relative to the terminal enzyme can be explained by the longer adaptation times that are possible for higher final times. Thus, if slower responses are sufficient (equalling higher final times), a stronger control of pathway flux through the initial enzyme is adequate.

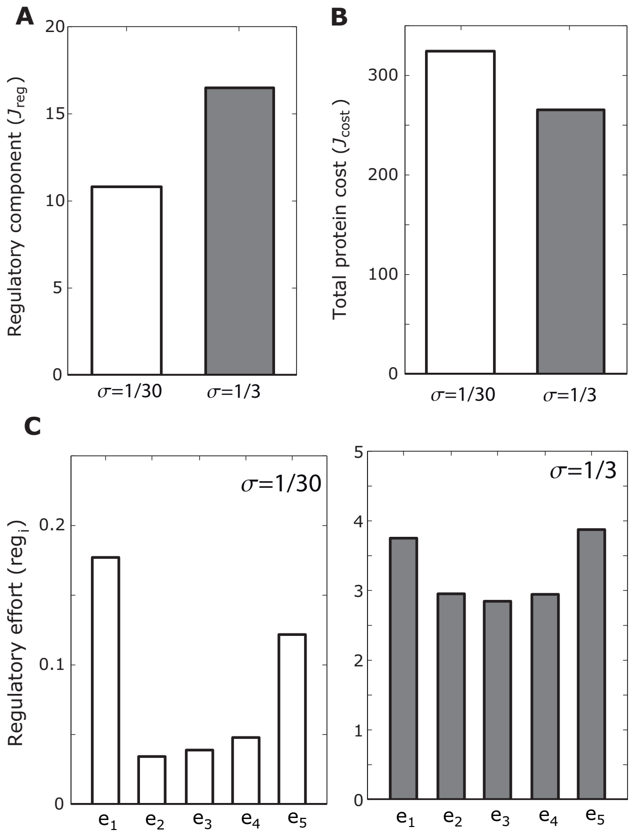

Figure S3: **Optimal pathway regulation for pathways with $k_{cat,i}$ and K_m values from *in vitro* data (Bar-Even *et al.* (2011))**. (**A**) Regulatory component (J_{reg}) for $\sigma = 1/30$ and for $\sigma = 1/3$. (**B**) total protein cost (J_{cost}) and regulatory efforts (reg_i), measured as absolute change in enzyme concentrations, at different pathway positions, (**C**) for $\sigma = 1/30$ and **D**) for $\sigma = 1/3$. For simulations, we determined the median value of $k_{cat,i}$ from the data of Bar-Even *et al.* (2011) and divided the experimentally measured $k_{cat,i}$ and K_m values by the median value (to preserve the relationship between $k_{cat,i}$ and K_m). Thus, the median value of all $k_{cat,i}$ subsequently was one. Scaling was necessary to avoid problems due to very large and small parameters during optimization. Additionally, we adjusted the concentration of the substrate of the pathway S to the median of the scaled K_m values to take into account that metabolite concentrations in most pathways are equal or larger than K_m values (Bennett *et al.*, 2009). Displayed data shows the median of 150 optimization runs with $k_{cat,i}$ and K_m values sampled from Bar-Even *et al.* (2011).

Modifications on the mathematical formulation to study the influence of protein biosynthetic rates

In order to study the influence of protein biosynthetic rates on pathway regulation the problem formulation was modified by adding constraints on the rate of change of enzyme concentrations. Mathematically this reads as:

$$\left| \frac{de_i(t)}{dt} \right| \leq m \quad for \quad i = 1:5 \tag{10}$$

As in Wessely *et al.* (2011) the aim of the optimization is to identify a time-course of the enzymes $e_i(t)$ that minimizes the objective function (Eq. 1). Subject to the system dynamics (Eq. 2) and according to changes in the outflow of the product (Eq. 3). The control variables have to obey constraints on the concentration of the product (Eq. 7) as well as the concentration of the intermediate metabolites (Eq. 8). Additionally the control variables are subject to the constraints on the rate of change (Eq. 10).

The optimal regulatory programs were calculated for three different rate of changes ($m = 0.06$ and 0.15). For each case 150 runs were performed, each with random kinetic parameters and dilution rates. Complete boxplots are presented in Figure S4. To obtain these results the control variables were approximated using 14 variable length linear elements. It should be noticed that PWL-v allows one to significantly reduce the number of decision variables. However, the introduction of switching times as decision variables increases the multimodality of the problem.

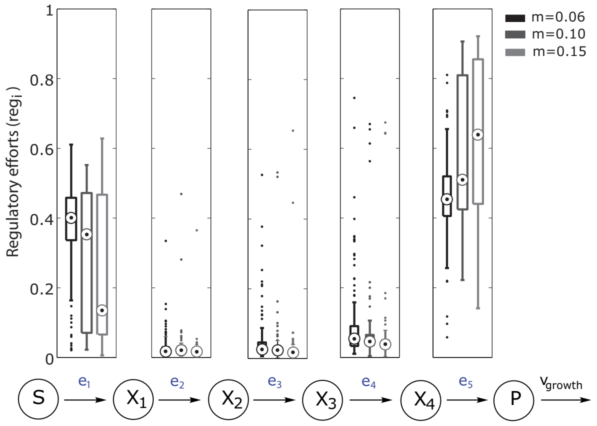

Figure S4: Regulatory efforts (reg_i), measured as absolute change in enzyme concentrations, at different pathway positions for constrained protein biosynthetic rates. Boxplots presents results for 150 optimization runs with randomized parameters and dilution values ($\sigma = 1/30$). Individual enzyme profiles were approximated using piece-wise linear interpolation with varying length elements. A low value of m corresponds to slow protein biosynthetic rates (black boxes) while high values corresponds to fast protein biosynthetic rates (grey boxes). Medians are indicated by circles.

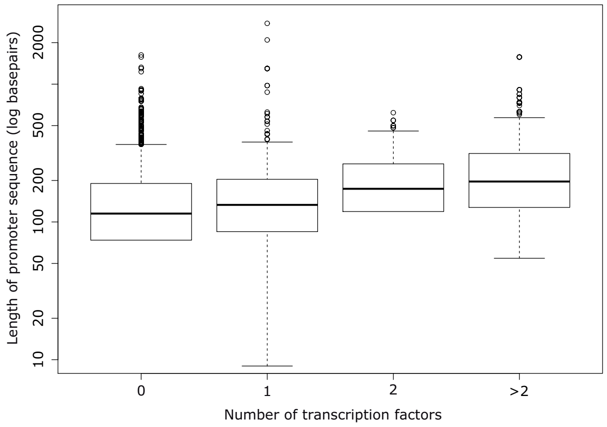

Figure S5: **Relationship between promoter lengths and number of controlling transcription factors.** Gene of *E. coli* were classified according to the number of transcription factors. For each class, box plots show the length of promoter regions in base pairs which were determined as described in the main manuscript. Please not the logarithmic scale of the y-axis.

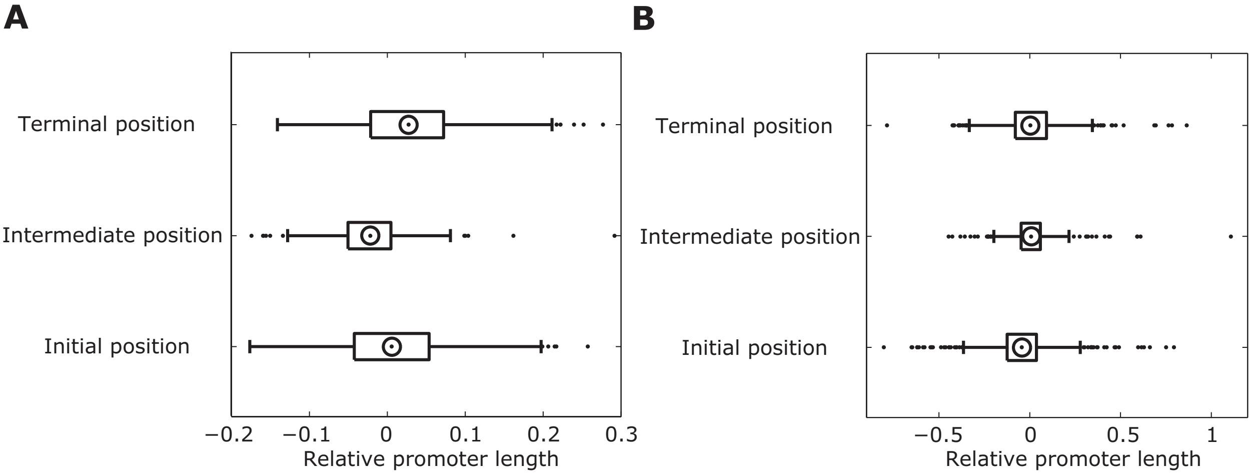

Figure S6: **Average lengths of upstream regions in sparsely and pervasively regulated metabolic pathways.** Average lengths of upstream regions at different pathway positions in **A** sparsely and **B** pervasively regulated metabolic pathways were determined. For the definition of sparsely and pervasively regulated metabolic pathways see the methods section of the main document. In sparsely regulated metabolic pathways, we find that there is an increase in the amount of transcriptional regulation at the initial and terminal position compared to intermediate positions (Wilcoxon test p-value=0 and $2.2 \cdot 10^{-9}$, respectively). For pervasively regulated pathways we find no significant differences in the length of upstream regions between initial and intermediate positions, but a significant decrease between intermediate and terminal position (Wilcoxon test p-value=0.59 and $1.22 \cdot 10^{-10}$). These analyses across all organisms closely follow results on the frequency of regulation in sparsely and pervasively regulated metabolic pathways in *Escherichia coli* (Wessely *et al.*, 2011). Data from 309 organisms with data from at least ten genes in initial, intermediate and terminal positions in a sparsely regulated pathway displayed in A and data from 520 organisms with data from at least ten genes in initial, intermediate and terminal positions in a pervasively regulated pathway displayed in B.

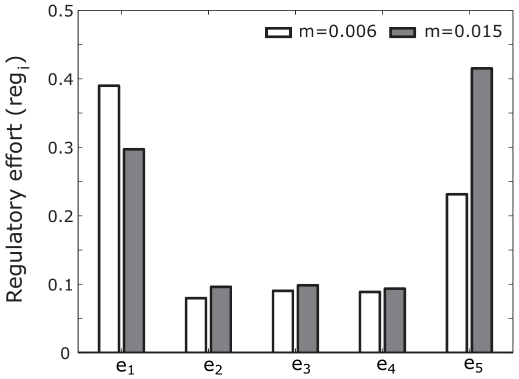

Figure S7: **Influence of time-scales between metabolite and transcriptional dynamics.** To study the influence of differences in time-scales between metabolite, transcriptional and growth dynamics, optimization runs were repeated with protein biosynthetic rates (Eq. 10) divided by ten and dilution values $[d_1, d_2, d_3]$ sampled from the interval $[0.02, 0.08]$ (for the original simulations, dilution values were sampled from $[0.2, 0.8]$). Additionally, the final time was increased to $t_f = 300$ due to the increased transcriptional response time. Bars represent median values (150 optimization runs with randomized parameters and dilution values ($\sigma = 1/30$)) for individual regulatory efforts, measured as absolute change in enzyme concentrations, at different pathway positions for constrained protein biosynthetic rates. A low value of m corresponds to slow protein biosynthetic rates (white boxes) while high values corresponds to fast protein biosynthetic rates (grey boxes). Also in this case we observe a decrease in regulatory effort at the first and an increase at the terminal enzyme for faster protein biosynthetic rates.

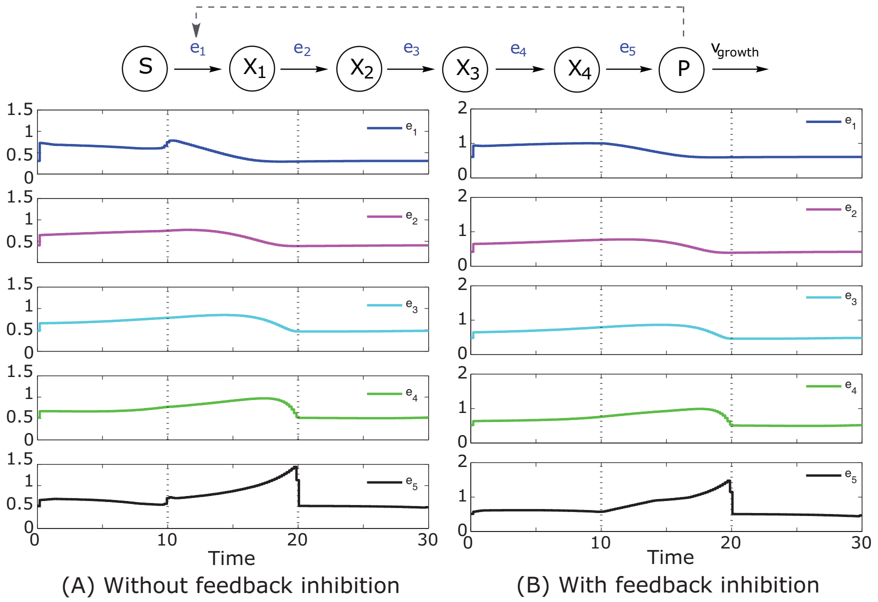

Figure S8: Optimal enzyme profiles for a weight cost $\sigma = 1/3$ for the linear chain (with unit kinetic parameters) case A, and for the case with feedback inhibition of e_1 by the product (case B). In both cases optimal profiles were calculated with a discretization level of ρ =160 steps with fixed length. The y-axis indicates concentration of enzymes and y-axis presents time (arbitrary units).

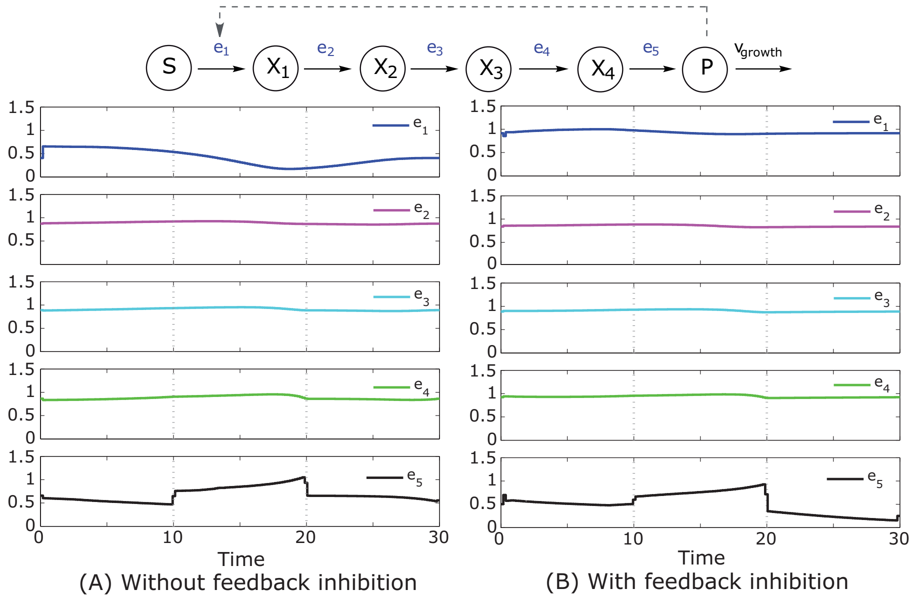

Figure S9: Optimal enzyme profiles for a weight cost $\sigma = 1/30$ for the linear chain (with unit kinetic parameters) case A, and for the case with feedback inhibition of e_1 by the product (case B). In both cases optimal profiles were acquired with a discretization level of ρ =160 steps with fixed length. The y-axis indicates concentration of enzymes and y-axis presents time (arbitrary units).

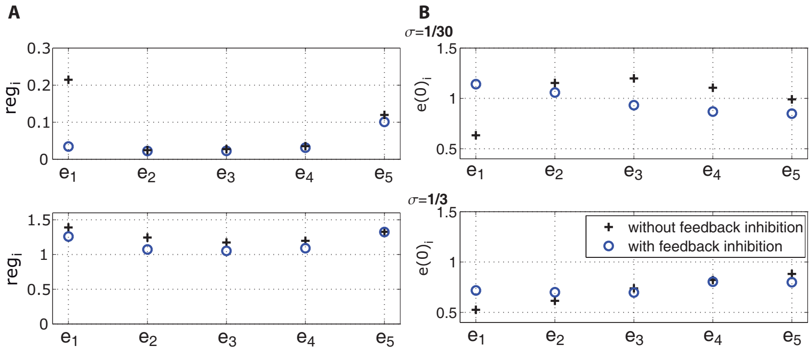

Figure S10: Regulatory efforts of individual enzymes (reg_i) (A) and changes in initial enzyme concentrations (B) for $\sigma = 1/30$, and $\sigma = 1/3$ with random kinetic constants and dilution rates. In general, the contribution of initial enzyme concentrations is increased across all σ values. The regulation of the initial enzyme is most strongly reduced for $\sigma = 1/30$ while it is marginally reduced for $\sigma = 1/3$. For pathways with lowly abundant proteins ($\sigma = 1/30$), there is a high frequency of post-translational regulatory events while the frequency of transcriptional regulatory events is increased but lower and less significant than for terminal enzymes. In contrast, in pathways with highly abundant proteins ($\sigma = 1/3$) there is a high frequency of transcriptional regulatory events even in the presence of post-translational regulation of the pathway.

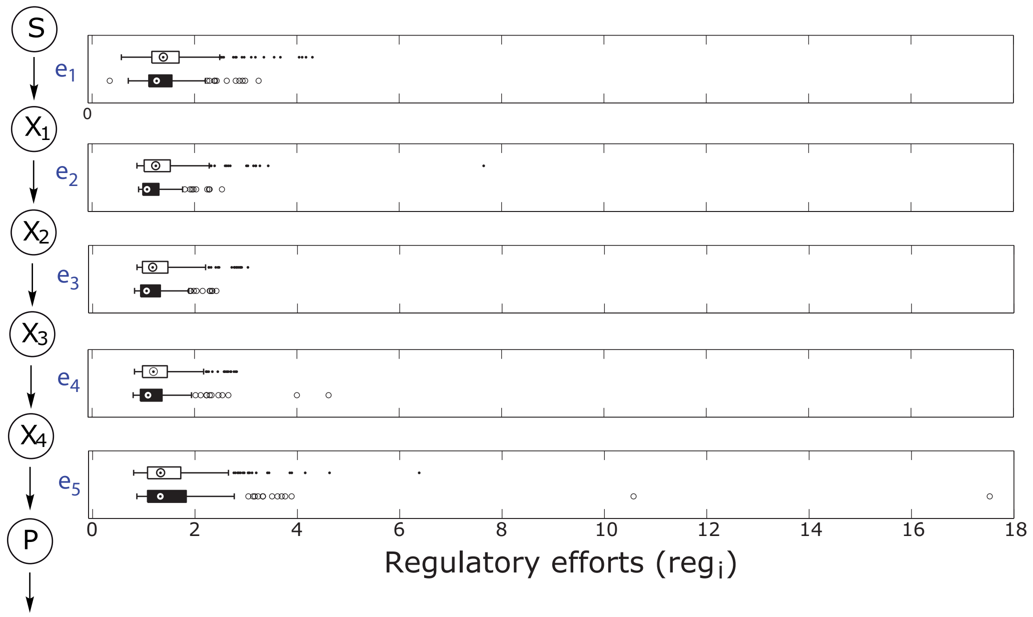

Figure S11: Reguralory efforts (reg_i), measured as absolute change in enzyme concentrations, for a weight cost $\sigma = 1/3$ (linear chain). Runs without inhibition depicted in white, runs with inhibition depicted with black boxes (two hundred optimizations with randomized parameters and dilution values for each case).

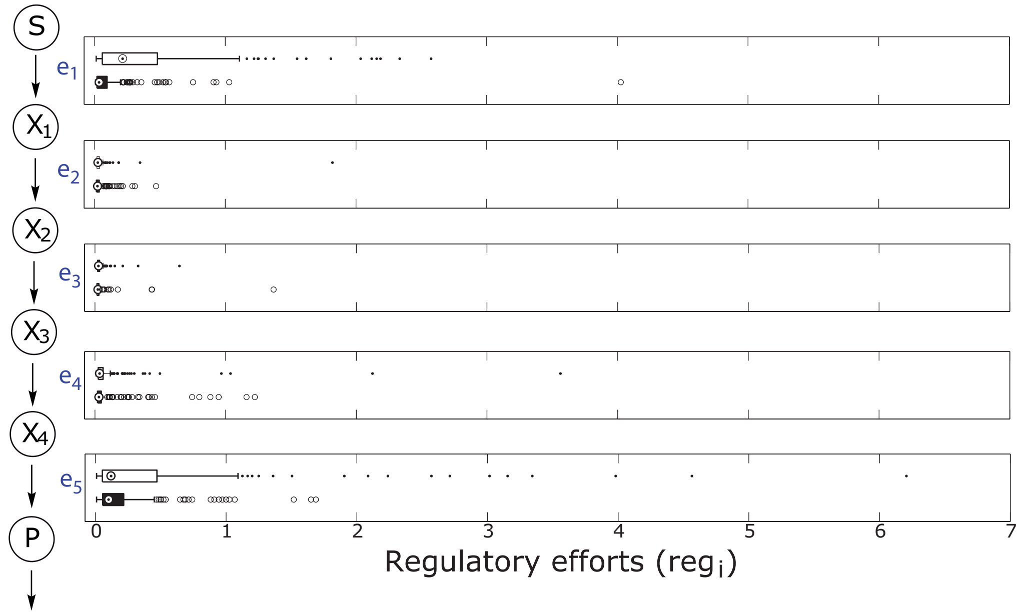

Figure S12: Regulatory efforts (reg_i), measured as absolute change in enzyme concentrations, for a weight cost $\sigma = 1/30$ (linear chain). Runs without inhibition depicted in white, runs with inhibition depicted with black boxes (two hundred optimizations with randomized parameters and dilution values for each case).

S3 Converging and branching pathways

Mathematical formulation of the pathway with a converging reaction

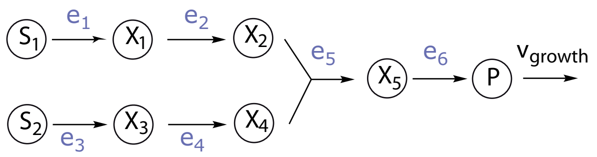

Figure S13: Scheme of the pathway with a converging branch where two substrates are converted into a product through six reactions following Michaelis-Menten kinetics.

Find **e**(t) over t ϵ $[t_0, t_f]$ to minimize:

$$\underbrace{min \sum_{i=1}^{6} \sigma \cdot e_i(0) \cdot t_f}_{J_{cost} = \sum_{i=1}^{6} cost_i} + \underbrace{\sum_{i=1}^{6} \int_{t_0=0}^{t_f=30} (e_i(t) - e_i(0))^2 dt}_{J_{reg} = \sum_{i=1}^{6} reg_i} \tag{11}$$

Subject to the system dynamics:

$$\begin{aligned} \frac{ds_1(t)}{dt} &= 0 \\ \frac{ds_2(t)}{dt} &= 0 \\ \frac{dx_1(t)}{dt} &= \nu_1(t) - \nu_2(t) \\ \frac{dx_2(t)}{dt} &= \nu_2(t) - \nu_5(t) \\ \frac{dx_3(t)}{dt} &= \nu_3(t) - \nu_4(t) \\ \frac{dx_4(t)}{dt} &= \nu_4(t) - \nu_5(t) \\ \frac{dx_5(t)}{dt} &= \nu_5(t) - \nu_6(t) \\ \frac{dp(t)}{dt} &= \nu_6(t) - \nu_{growth}(t) \end{aligned} \tag{12}$$

Where:

$$v_1(t) = \frac{k_{cat,1} \cdot s_1(t)}{K_{m,1} + s_1(t)} \cdot e_1(t) \tag{13}$$

$$v_3(t) = \frac{k_{cat,3} \cdot s_2(t)}{K_{m,3} + s_2(t)} \cdot e_3(t) \tag{14}$$

$$v_5(t) = \frac{k_{cat,5} \cdot x_2(t) \cdot x_4(t)}{K_{x_2,1} \cdot K_{x_4,2} + K_{x_4,2} \cdot x_2(t) + K_{x_2,2} \cdot x_4(t) + x_2(t) \cdot x_4(t)} \cdot e_5(t) \tag{15}$$

$$v_i(t) = \frac{k_{cat,i} \cdot x_{i-1}(t)}{K_{m,i} + x_{i-1}(t)} \cdot e_i(t) \tag{16}$$

$$v_{growth}(t) = \begin{cases} d_1 & \text{if } t < 10 \\ d_2 & \text{if } 10 \leq t \leq 20 \\ d_3 & \text{if } 20 \leq t \leq 30 \end{cases}$$

And the following path constraints:

$$0.8 \leq p(t) \leq 1.2 \quad (17)$$

$$x_1(t) + x_2(t) + x_3(t) + x_4(t) + x_5(t) \leq \Omega \quad (18)$$

$$e_i(t) \geq 0 \quad (19)$$

with:$\sigma = 1/30$, $\Omega = 5$, $s_1(t_0) = 1$, $s_2(t_0) = 1$, $x_i(t_0) = 1$, $p(t_0) = 1$, $[d_1, d_2, d_3]\epsilon[0.2, 0.8]$ and all the kinetic parameters ($k_{cat,1}$, $K_{m,1}$, $k_{cat,i}$, $K_{m,i}$ $K_{x_2,1}$,$K_{x_4,2}$ and $K_{x_2,2}$) $\epsilon[0, 2]$.

Mathematical formulation of a divergent branch

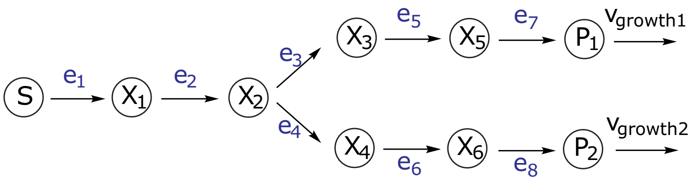

Figure S14: Scheme of the pathway with a converging branch considered in this example.

Find $\mathbf{e}$(t) over t ϵ $[t_0$,$t_f]$ to minimize:

$$\underbrace{min \sum_{i=1}^{8} \sigma \cdot e_i(0) \cdot t_f}_{J_{cost} = \sum_{i=1}^{8} cost_i} + \underbrace{\sum_{i=1}^{8} \int_{t_0=0}^{t_f=30} (e_i(t) - e_i(0))^2 dt}_{J_{reg} = \sum_{i=1}^{8} reg_i} \quad (20)$$

Subject to the system dynamics:

$$\begin{aligned}
\frac{ds_1(t)}{dt} &= 0 \\
\frac{dx_1(t)}{dt} &= \nu_1(t) - \nu_2(t) \\
\frac{dx_2(t)}{dt} &= \nu_2(t) - \nu_3(t) - \nu_4(t) \\
\frac{dx_3(t)}{dt} &= \nu_3(t) - \nu_5(t) \\
\frac{dx_4(t)}{dt} &= \nu_4(t) - \nu_6(t) \\
\frac{dx_5(t)}{dt} &= \nu_5(t) - \nu_7(t) \\
\frac{dx_6(t)}{dt} &= \nu_6(t) - \nu_8(t) \\
\frac{dp_1(t)}{dt} &= \nu_7(t) - \nu_{growth1}(t)
\end{aligned}$$

$$\frac{dp_2(t)}{dt} = \nu_8(t) - \nu_{growth2}(t) \tag{21}$$

Where:

$$\upsilon_1(t) = \frac{k_{cat,1} \cdot s(t)}{K_{m,1} + s(t)} \cdot e_1(t) \tag{22}$$

$$\upsilon_i(t) = \frac{k_{cat,i} \cdot x_{i-1}(t)}{K_{m,i} + x_{i-1}(t)} \cdot e_i(t) \quad for \quad i = 2:3 \tag{23}$$

$$\upsilon_i(t) = \frac{k_{cat,i} \cdot x_{i-2}(t)}{K_{m,i} + x_{i-2}(t)} \cdot e_i(t) \quad for \quad i = 4:8 \tag{24}$$

And the following path constraints:

$$0.8 \leq p_i(t) \leq 1.2 \quad for \quad i = 1:2 \tag{25}$$

$$x_1(t) + x_2(t) + x_3(t) + x_4(t) + x_5(t) + x_6(t) \leq \Omega \tag{26}$$

$$e(t) \geq 0 \tag{27}$$

The problem was solved using the following values of the dilution rates:

$$\upsilon_{growth1}(t) = \begin{cases} d_1 & \text{if } t < 10 \\ d_2 & \text{if } 10 \leq t \leq 20 \\ d_3 & \text{if } 20 \leq t \leq 30 \end{cases}$$

$$\upsilon_{growth2}(t) = \begin{cases} d_4 & \text{if } t < 10 \\ d_5 & \text{if } 10 \leq t \leq 20 \\ d_6 & \text{if } 20 \leq t \leq 30 \end{cases}$$

with:$\sigma = 1/30$, $\Omega = 6$, $s(t_0) = 1$, $x_i(t_0) = 1$, $p_1(t_0) = 1$, $p_2(t_0) = 1$, $[d_1, d_2, d_3, d_4, d_5, d_6]\epsilon[0.2, 0.8]$ and for $i = 1:8$ $k_{cat,i}$ and $K_{m,i}$ $\epsilon[0, 2]$.

Mathematical formulation for the problems with feedback inhibition over different positions of the pathway

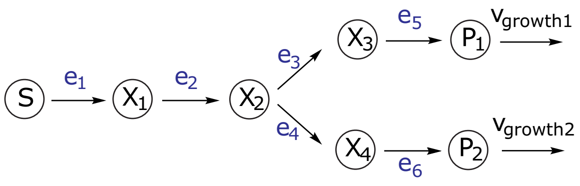

Figure S15: Scheme of the pathway with a converging branch considered in this example.

The problem formulation for the branched pathway presented in Figure S15 is as follows. Find **e**(t) over t ϵ $[t_0, t_f]$ to minimize:

$$min \underbrace{\sum_{i=1}^{6} \sigma \cdot e_i(0) \cdot t_f}_{J_{cost}=\sum_{i=1}^{6} cost_i} + \underbrace{\sum_{i=1}^{6} \int_{t_0=0}^{t_f=30} (e_i(t) - e_i(0))^2 dt}_{J_{reg}=\sum_{i=1}^{6} reg_i} \tag{28}$$

Subject to the system dynamics:

$$\begin{aligned}
\frac{ds_1(t)}{dt} &= 0 \\
\frac{dx_1(t)}{dt} &= \nu_1(t) - \nu_2(t) \\
\frac{dx_2(t)}{dt} &= \nu_2(t) - \nu_3(t) - \nu_4(t) \\
\frac{dx_3(t)}{dt} &= \nu_3(t) - \nu_5(t) \\
\frac{dx_4(t)}{dt} &= \nu_4(t) - \nu_6(t) \\
\frac{dp_1(t)}{dt} &= \nu_5(t) - \nu_{growth1}(t) \\
\frac{dp_2(t)}{dt} &= \nu_6(t) - \nu_{growth2}(t)
\end{aligned} \tag{29}$$

Where:

$$\upsilon_1(t) = \frac{k_{cat,1} \cdot s(t)}{K_{m,1} + s(t)} \cdot e_1(t) \tag{30}$$

$$\upsilon_i(t) = \frac{k_{cat,i} \cdot x_{i-1}(t)}{K_{m,i} + x_{i-1}(t)} \cdot e_i(t) \quad for \quad i = 2:3 \tag{31}$$

$$\upsilon_i(t) = \frac{k_{cat,i} \cdot x_{i-2}(t)}{K_{m,i} + x_{i-2}(t)} \cdot e_i(t) \quad for \quad i = 4:6 \tag{32}$$

$$\upsilon_{growth1}(t) = \begin{cases} d_1 & \text{if } t < 10 \\ d_2 & \text{if } 10 \le t \le 20 \\ d_3 & \text{if } 20 \le t \le 30 \end{cases}$$

$$\upsilon_{growth2}(t) = \begin{cases} d_4 & \text{if } t < 10 \\ d_5 & \text{if } 10 \le t \le 20 \\ d_6 & \text{if } 20 \le t \le 30 \end{cases}$$

And the following path constraints:

$$0.8 \le p_i(t) \le 1.2 \quad for \quad i = 1:2 \tag{33}$$

$$x_1(t) + x_2(t) + x_3(t) + x_4(t) \leq \Omega \tag{34}$$

$$e(t) \geq 0 \tag{35}$$

with: $\sigma = 1/30$, $\Omega = 4$, $s(t_0) = 1$, $x_i(t_0) = 1$, $p_1(t_0) = 1$, $p_2(t_0) = 1$, $[d_1, d_2, d_3, d_4, d_5, d_6] \epsilon [0.2, 0.8]$ and for $i = 1:6$ $k_{cat,i}$ and $K_{m,i}$ $\epsilon [0, 2]$

Mathematical formulation of the double feedback inhibition over the initial position

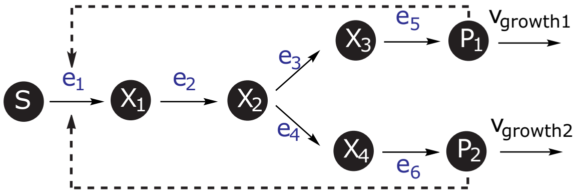

Figure S16: Representation of the converging pathway with feedback inhibition over the initial step by the two products.

The model presented in previous section was modified in order to incorporate feedback inhibition from the two different products of the pathway over the initial step (green dashed line in Figure S16). Flux through reaction one is now represented by Eq. 36, the rest of the problem formulation remains unchanged.

$$v_1(t) = \frac{k_{cat,1} \cdot s(t)}{s(t) + K_{m,1}\left(1 + \frac{I_1(t)}{k_{r,1}}\right)\left(1 + \frac{I_2(t)}{k_{r,2}}\right)} \cdot e_1(t) \tag{36}$$

where $I_1(t)$ and $I_2(t)$ are the inhibitors in this case the products ($p_1(t)$ and $p_2(t)$, respectively), $k_{r,1}$ and $k_{r,2}$ corresponds to the strength of the feedback inhibition.

Mathematical formulation of double feedback inhibition to the pathway branches

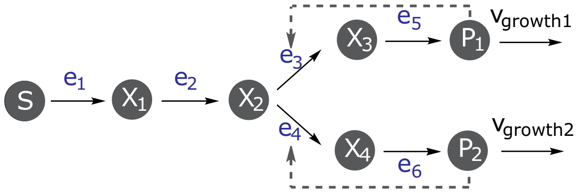

Figure S17: Representation of a converging pathway with feedback inhibition from the products ($p_1(t)$ and $p_2(t)$) over the branching enzymes (blue dashed line).

In this case (Figure S17), the flux through reactions three and four are represented by Eq. 37 and 38, the rest of the problem formulation remains unchanged.

$$v_3(t) = \frac{k_{cat,3} \cdot x_2(t)}{x_2(t) + K_{m,3}\left(1 + \frac{I_1(t)}{k_{r,3}}\right)} \cdot e_3(t) \tag{37}$$

$$v_4(t) = \frac{k_{cat,4} \cdot x_3(t)}{x_3(t) + K_{m,4}\left(1 + \frac{I_2(t)}{k_{r,4}}\right)} \cdot e_4(t) \tag{38}$$

where $I_1(t)$ and $I_2(t)$ are the inhibitors, in this case the products ($p_1(t)$ and $p_2(t)$), e_3 and e_4 are the targets of inhibition, respectively. $k_{r,3}$ and $k_{r,4}$ correspond to the strength of the feedback inhibition.

Mathematical formulation of feedback inhibition over initial and branching reactions

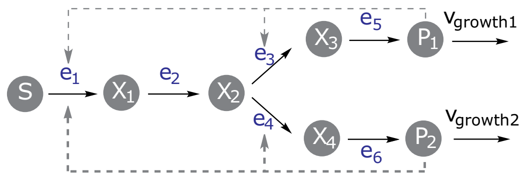

Figure S18: Scheme of the converging pathway with inhibition over the initial step and the branching reactions by the products (red dashed line).

The two situations considered before were combined (Figure S18), now flux through the initial reaction is represented by Eq. 39 and flux through reactions three and four are represented by Eqs. 40 and 41.

$$\upsilon_1(t) = \frac{k_{cat,1} \cdot e_1(t) \cdot s(t)}{s(t) + K_m \left(1 + \frac{p_1(t)}{k_{r,1}}\right)\left(1 + \frac{p_2(t)}{k_{r,2}}\right)} \tag{39}$$

$$\upsilon_3(t) = \frac{k_{cat,3} \cdot e_3(t) \cdot x_2(t)}{x_2(t) + K_{m,3} \left(1 + \frac{p_1(t)}{k_{r,3}}\right)} \tag{40}$$

$$\upsilon_4(t) = \frac{k_{cat,4} \cdot e_4(t) \cdot x_3(t)}{x_3(t) + K_{m,4} \left(1 + \frac{p_2(t)}{k_{r,4}}\right)} \tag{41}$$

Complete boxplots in branched pathways

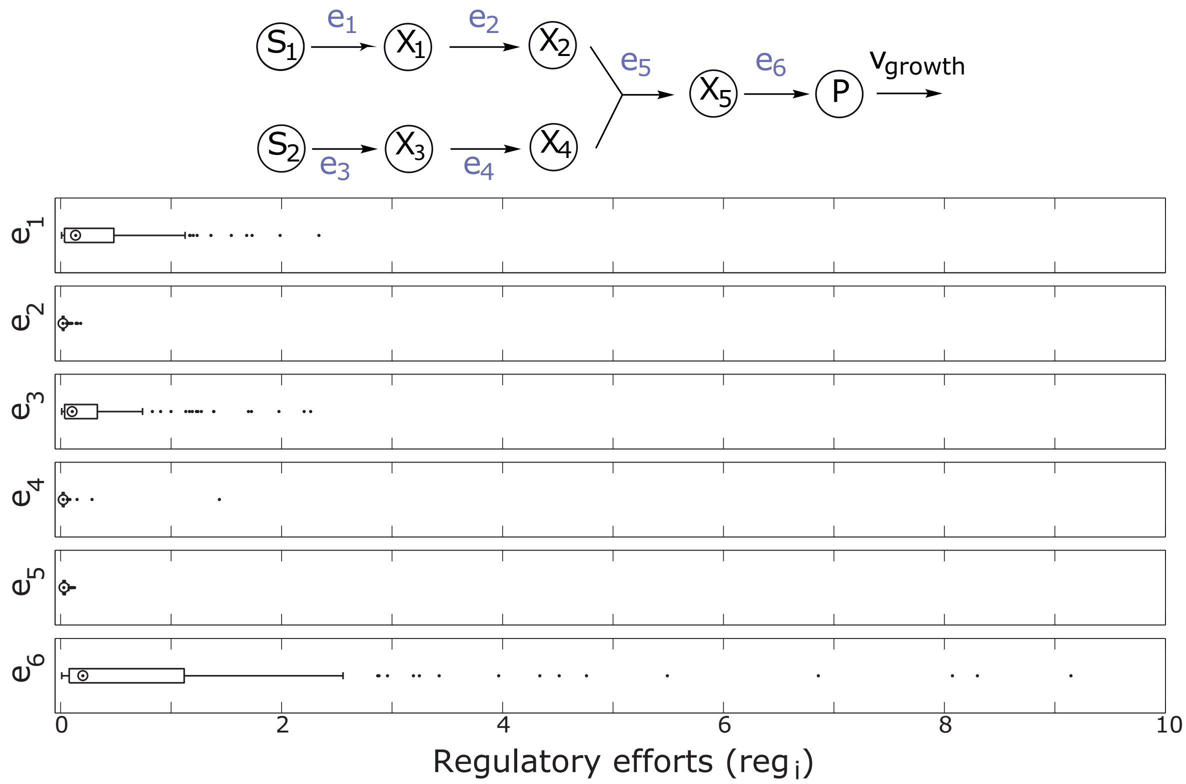

Figure S19: Complete boxplot for the regulatory efforts of individual enzymes (reg_i), measured as absolute change in enzyme concentration, in pathways with a converging reaction ($\sigma = 1/30$). Two hundred optimizations with randomized parameters and dilution values were performed. Individual optimizations were obtained with a discretization level of 160 steps with fixed length. There was almost no regulation at intermediate positions while most of the regulation was observed in the initial and terminal position of the pathway.

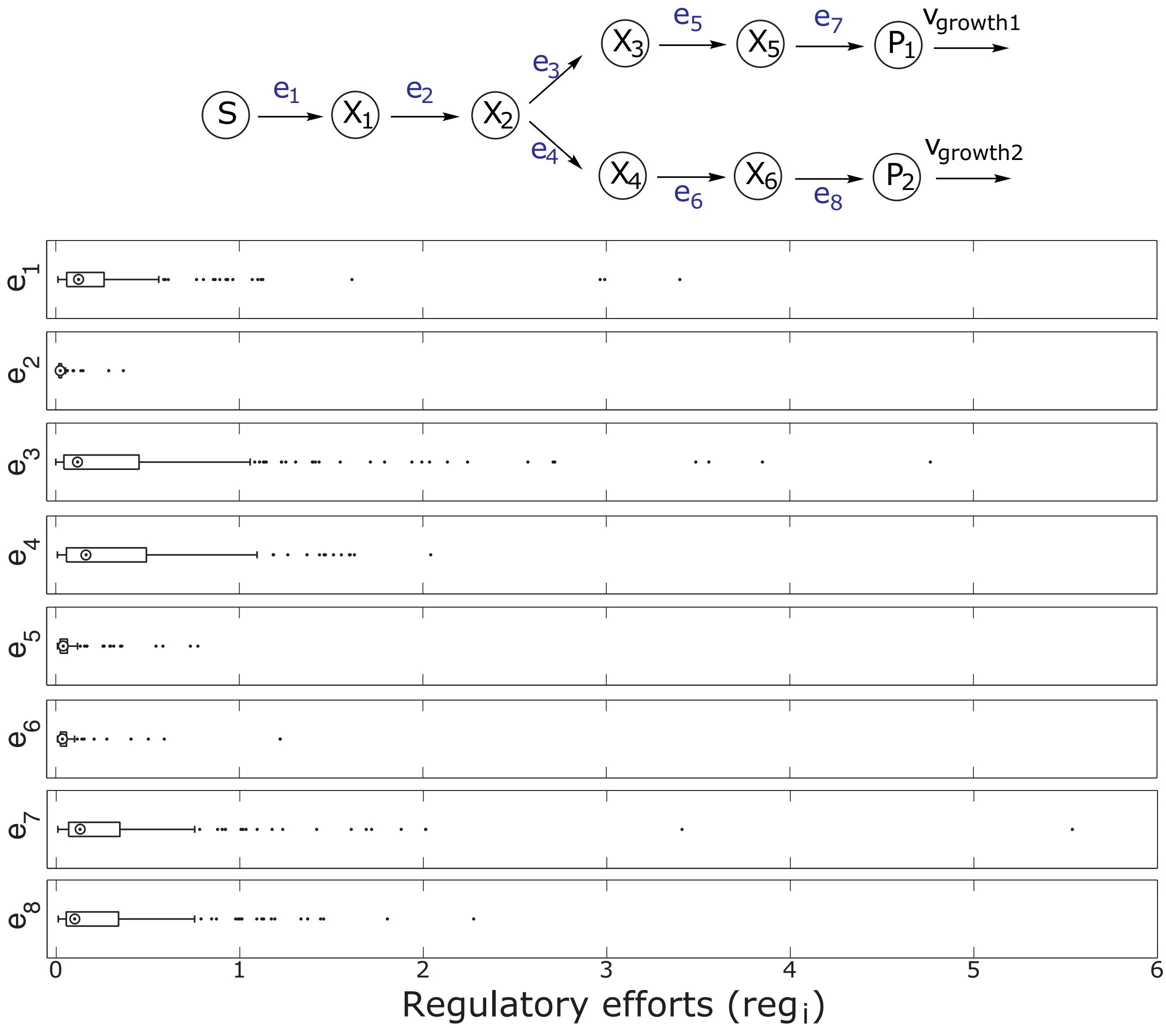

Figure S20: Complete boxplot for the regulatory efforts of individual enzymes (reg_i) for pathways with a divergent reaction ($\sigma = 1/30$) over two hundred optimizations with randomized parameters and dilution values. The regulatory effort was measured as absolute change in enzyme concentration. Apart from the regulation on the initial and terminal reactions we found that the enzymes after the pathway branch (e_3,e_4) were frequently regulated.

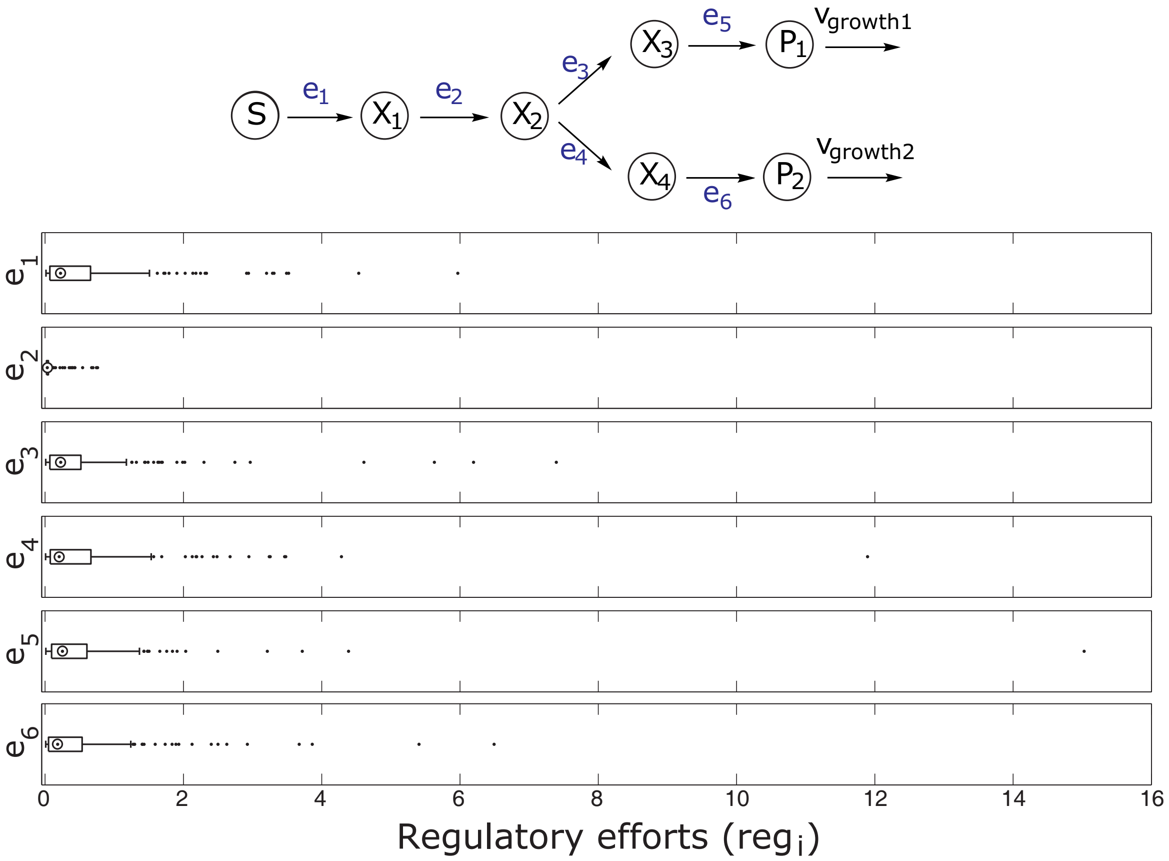

Figure S21: Complete boxplot for the regulatory efforts of individual enzymes (reg_i) for pathways with a divergent reaction (scheme presented above) over two hundred optimizations with randomized parameters and dilution values. The regulatory effort was measured as absolute change in enzyme concentration. Apart from the regulation on the initial and terminal reactions we found that the enzymes after the pathway branch (e_3, e_4) were frequently regulated.

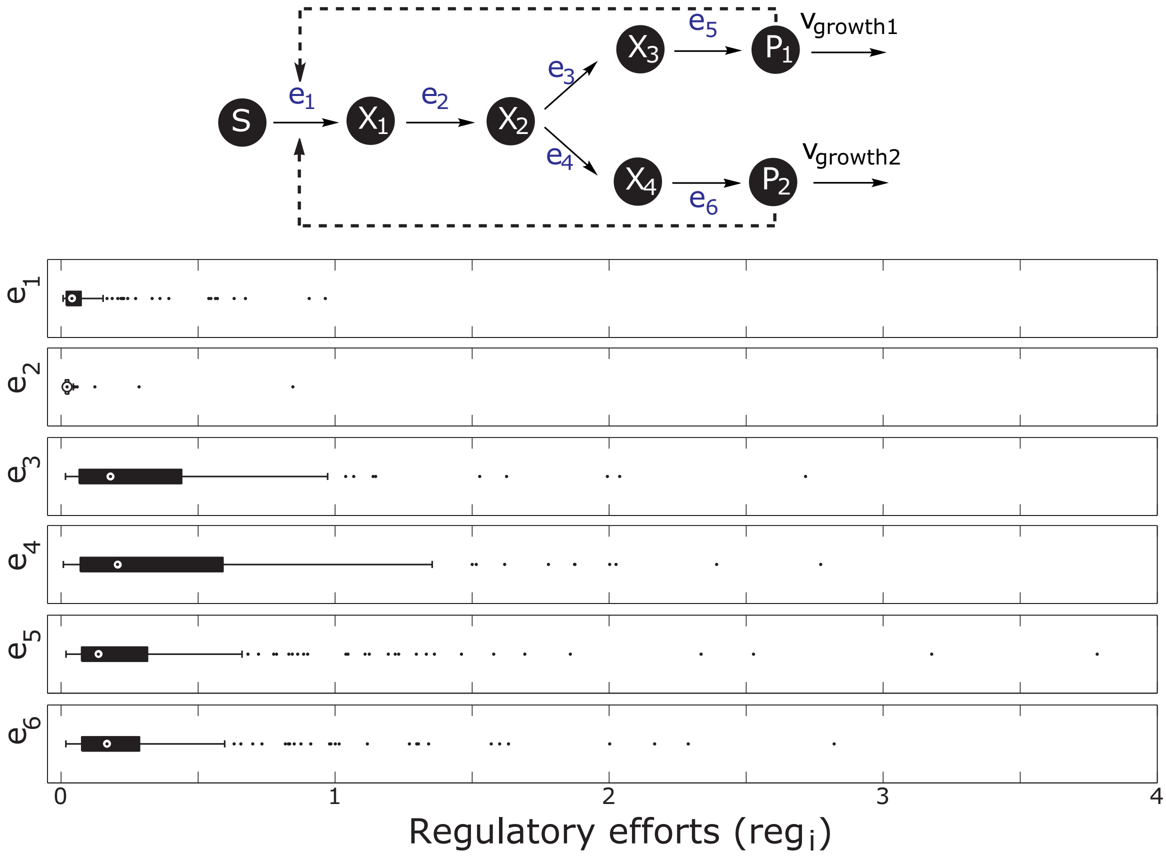

Figure S22: Regulatory efforts of individual enzymes (reg_i), measured as absolute change in enzyme concentration, when inhibition of the initial step of a pathway by the two products was considered ($\sigma = 1/30$). Boxplots shows the results of two hundred optimizations with randomized parameters and dilution values. We observed a drastic decrease on the regulatory effort in the initial position while branchpoint (e_3, e_4) and terminal reactions (e_5, e_6) continue to be regulated.

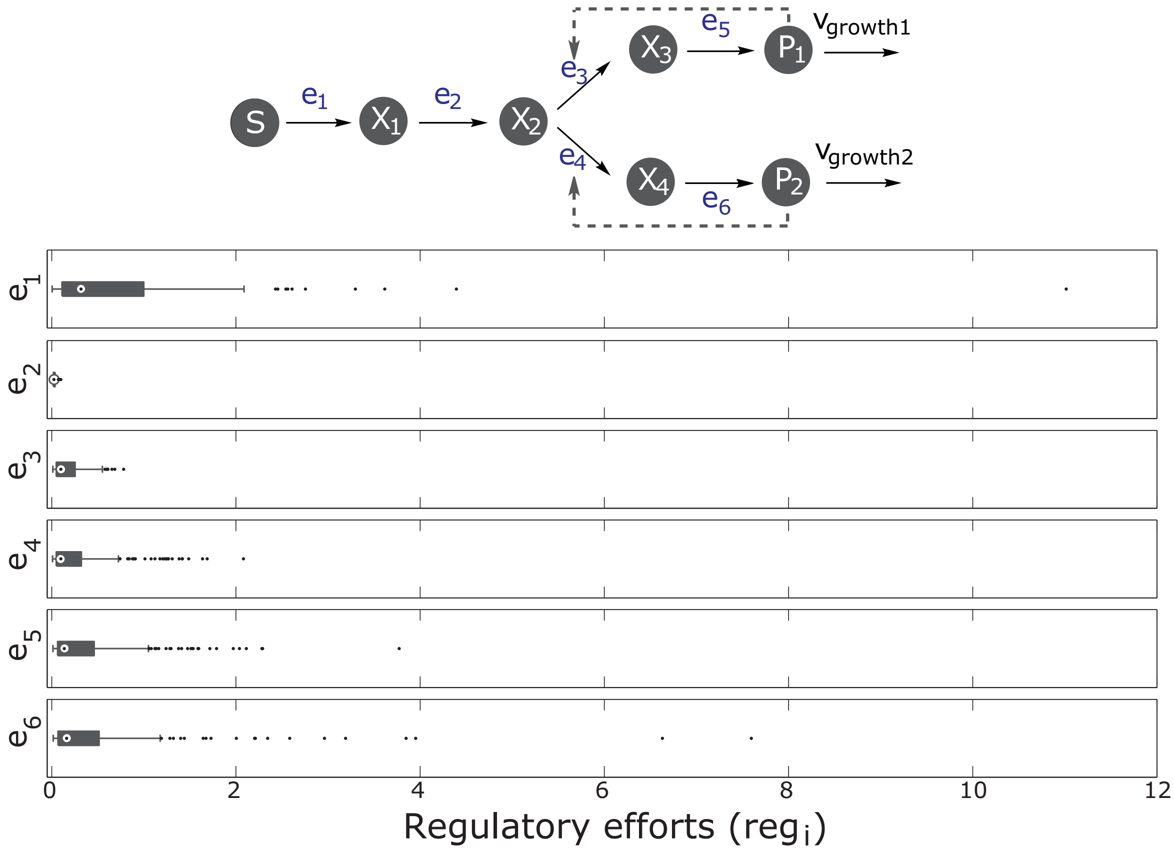

Figure S23: Complete boxplot (two hundred optimizations with randomized parameters and dilution values) for the regulatory efforts (reg_i) for the case where inhibition of the branching enzymes (e_3, e_4) by the products was considered ($\sigma = 1/30$). The regulatory effort was measured as absolute change in enzyme concentration. There was a decrease on the regulatory effort of the reaction after the branch but less meaningful that in the cases where inhibition of the initial step of a pathway by the two products was studied. Regulation in initial and terminal reactions was not affected.

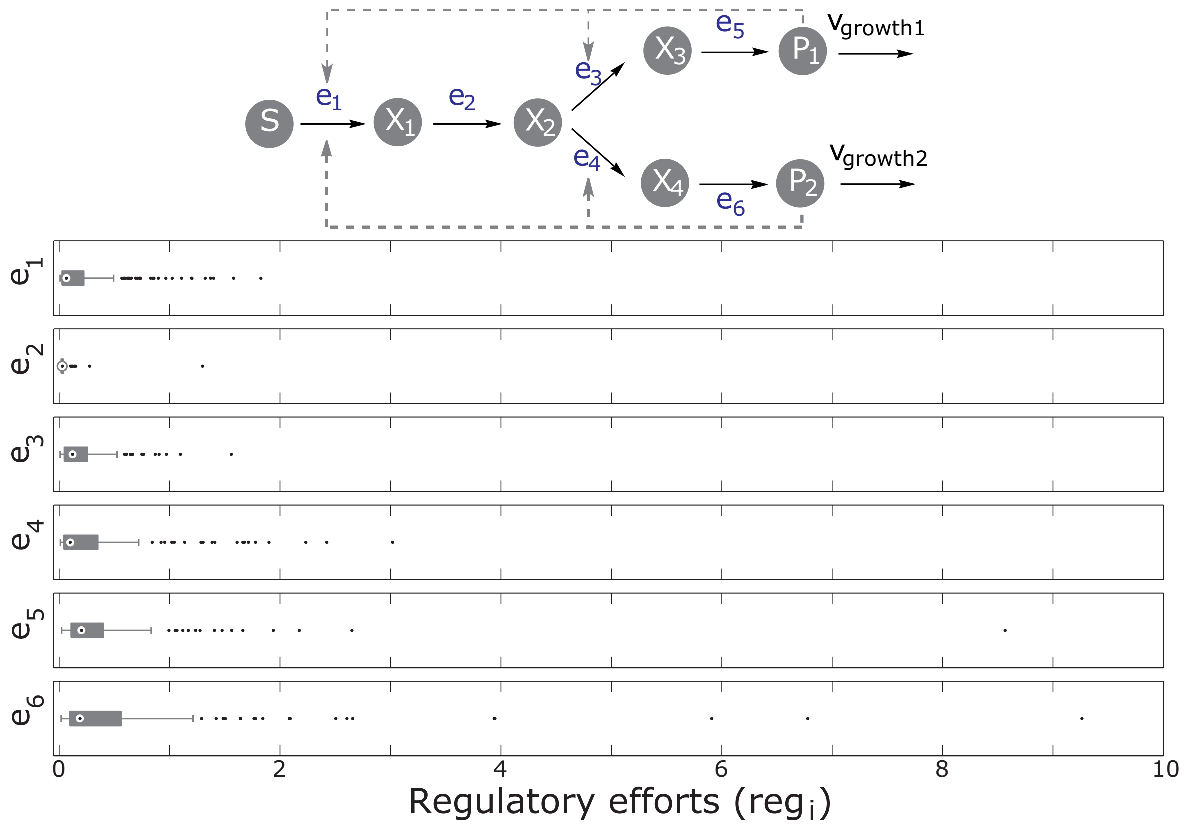

Figure S24: Regulatory efforts of individual enzymes (reg_i), measured as absolute change in enzyme concentration, when inhibition simultaneous inhibition of e_1, e_3 and e_4 by the two products was studied (for a weight cost of $\sigma = 1/30$). Boxplots presents the results of two hundred optimizations with randomized parameters and dilution values. We observed a drastic decrease on the regulatory effort in the initial position while branchpoint (e_3, e_4) and terminal reactions (e_5, e_6) continue to be regulated.

References

Balsa-Canto, E., Banga, J. R., Alonso, A. A., and Vassiliadis, V. S. (2001). Dynamic optimization of chemical and biochemical processes using restricted second-order information. *Comp & Chem Eng*, **25**, 539–546.

Balsa-Canto, E., Vassiliadis, V. S., and Banga, J. R. (2005). Dynamic optimization of single- and multi-stage systems using a hybrid stochastic-deterministic method. *Ind Eng Chem Res*, **44**(5), 1514–1523.

Banga, J. R., Balsa-Canto, E., Moles, C., and Alonso, A. A. (2005). Dynamic optimization of bioprocesses: Efficient and robust numerical strategies. *J Biotechnol*, **117**, 407–419.

Bar-Even, A., Noor, E., Savir, Y., Liebermeister, W., Davidi, D., Tawfik, D. S., and Milo, R. (2011). The Moderately Efficient Enzyme: Evolutionary and Physicochemical Trends Shaping Enzyme Parameters. *Biochemistry*, **50**(21), 4402–4410.

Bennett, B. D., Kimball, E. H., Gao, M., Osterhout, R., Van Dien, S. J., and Rabinowitz, J. D. (2009). Absolute metabolite concentrations and implied enzyme active site occupancy in *Escherichia coli*. *Nature Chemical Biology*, **5**(8), 593–599.

Biegler, L. T., Cervantes, A. M., and Wätcher, A. (2002). Advances in simultaneous strategies for dynamic process optimization. *Chem Eng Sci*, **57**(4), 575–593.

Bock, H. G. and Plitt, K. J. (1984). A multiple shooting algorithm for direct solution of optimal control problems. In *Proceedings 9th IFAC World Congress*, pages 242–247. Pergamon Press: New York.

Egea, J. A., Balsa-Canto, E., Garcia, M. G., and Banga, J. R. (2009). Dynamic optimization of nonlinear processes with an enhanced scatter search method. *Ind Eng Chem Res*, **48**(9), 4388–4401.

Hairer, E. and Wanner, G. (1996). *Solvind Ordinary Differential Equations II: Stiff and Differential Algebraic Problems*. Springer-Verlag.

Vassiliadis, V. S., Sargent, R. W. H., and Pantelides, C. C. (1994). Solution of a class of multistage dynamic optimization problems. 1. problems without path constraints. *Ind Eng Chem Res*, **33**(9), 2111–2122.

Wessely, F., Bartl, M., Guthke, R., Li, P., Schuster, S., and Kaleta, C. (2011). Optimal regulatory strategies for metabolic pathways in *E. coli* depending on protein costs. *Mol Syst Biol*, **7**, 515–528.

Zhou, J. L., Tits, A. L., and Lawrence, C. T. (1997). User's guide for FFSQP version 3.7: A fortran code for solving optimization programs, possibly minimax, with general inequality contraints and linear equality constraints, generating feasible iterates. Technical Report SRC-TR-92-107r5, Institute for systems research, University of Maryland.
